# Analysis of drug-drug interactions in spontaneous adverse drug reaction reports from EudraVigilance focusing on psychiatric drugs and somatic medication

**DOI:** 10.1186/s12888-025-07352-8

**Published:** 2025-10-02

**Authors:** Diana Dubrall, Rebecca Weber, Miriam Böhme, Matthias Schmid, Bernhardt Sachs, Catharina Scholl

**Affiliations:** 1 Institute for Medical Biometry, Informatics and Epidemiology, University Hospital Bonn, Venusberg-Campus 1, 53127 Bonn, Germany; 2 Research Division, Federal Institute for Drugs and Medical Devices (BfArM), Kurt-Georg-Kiesinger-Allee 3, 53175 Bonn, Germany; 3 Department for Dermatology and Allergy, University Hospital RWTH, Morillenhang 27, 52074 Aachen, Germany

**Keywords:** Drug-drug interactions, Spontaneous reports, Adverse drug reaction reports, Psychiatric drugs, Spontaneous reporting systems, Psychopharmacoepidemiology

## Abstract

**Background:**

Psychiatric diseases are often treated with several drugs. In addition, the risk of developing somatic comorbidities which may require drug therapy is higher in patients with than in patients without psychiatric diseases. Further on, the risk of drug-drug interactions (DDI) increases with the number of drugs taken. The aim of this study was to analyze whether already known DDI between psychiatric drugs and somatic medications still occur in everyday clinical practice.

**Methods:**

We analyzed 9,276 spontaneous adverse drug reaction (ADR) reports from Germany contained in the European ADR database EudraVigilance received between 2017 and 2021 for adults in which antidepressants, antipsychotics and mood stabilizers were reported as suspected/interacting. The ABDATA (a division of Avoxa—Mediengruppe Deutscher Apotheker GmbH) drug information system was used to identify already known potential DDI (pDDI) related to psychiatric drugs and somatic medications. An individual assessment of reports was performed to detect whether the pDDI actually occurred.

**Results:**

1,271 reports (49.7%) with 2,655 pDDI relating to 728 different potentially interacting drug pairs of psychiatric drugs and somatic medications were identified. Patients in reports with pDDI were older than patients in reports without pDDI (median 64 vs. 49 years) (*p* < 0.05). In addition, the number of pDDI and the number of drugs reported increased with rising age. Restricted to potentially interacting drugs pairs with more than 10 reports (*n* = 704 reports, 1,111pDDI), the two most frequently identified pDDIs were (i) hyponatremias related to antidepressants and diuretics (*n* = 362, 32.6%) and (ii) bleeding events related to selective serotonin reuptake inhibitors (SSRI) and platelet aggregation inhibitors, anticoagulants or non-steroidal anti-inflammatory drugs (NSAIDs) (*n* = 295, 26.5%). After individual assessment of reports, bleeding events were found in 33.3% (14/42), 23.7% (45/190) and 17.4% (8/46) of reports related to SSRIs and anticoagulants, SSRIs and platelet aggregation inhibitors and SSRIs and NSAIDs. Hyponatremia was observed in 7.6% (22/289) of the reports related to antidepressants and diuretics.

**Conclusions:**

Well-known DDI still occur to a considerable amount in everyday clinical practice in psychiatric patients treated with psychiatric drugs and somatic medications. Special attention should be paid to older polymedicated patients.

**Supplementary Information:**

The online version contains supplementary material available at 10.1186/s12888-025-07352-8.

## Introduction

Drug–Drug-Interactions (DDI) are a well-known cause of adverse drug reactions (ADR). Even if not all potential DDI (pDDI) result in an ADR, these ADRs may be preventable by avoiding certain drug combinations or by appropriate dosage adjustments. It is familiar that the risk of a DDI increases with the number of drugs taken [1]. Therefore, recognizing DDI is of special interest in an aging society and certain high-risk patient groups like psychiatric patients who are frequently exposed to polymedication.

In a recent study analyzing polymedication in psychiatric inpatients, one third of the analyzed patients received at least five drugs concomitantly [2]. Treatment regimens in which patients are treated with combinations of multiple antidepressants, antipsychotics, mood stabilizers, anxiolytics, hypnotics, antihistamines, and anticholinergics are becoming more common, especially in psychiatric patients with severe mental illness (SMI) [3–5].

Furthermore, somatic comorbidities increase the need for additional medications in SMI patients. Around fifty percent of patients with psychiatric disorders suffer from a clinically relevant somatic comorbidity requiring treatment, e.g. cardiovascular disease, diabetes mellitus, and pulmonary disease. The risk of somatic diseases is twice as high in SMI patients as in individuals without a psychiatric disorder [6, 7]. This fact may be attributed to an interdependence between psychiatric and somatic disorders. In addition, ADRs to psychiatric drugs (e.g. weight gain or cardiovascular ADRs) are common and may increase the likelihood of developing somatic comorbidities [8]. At the same time patients with specific or several somatic diseases carry a higher risk of developing a psychiatric disorder [9]. Adequate treatment of somatic comorbidities is therefore of utmost importance. In hospital psychiatry DDI continue to occur frequently in SMI patients [2] and may be under-recognized by physicians and psychiatrists.

ADR databases containing ADR reports spontaneously reported in everyday clinical practice are an established source for identifying post-marketing safety signals [10]. In addition, they are also suitable for identifying and characterizing DDI. Several recent studies used spontaneous ADR reports to analyze DDI [11–16], demonstrating the feasibility of such an approach. In these studies, national or regional ADR databases from different countries were investigated, with exception of the analysis by Hult et al. who used the WHO global database Vigibase [11]. Notably, pDDI were detected in about 30–40% of the ADR reports [12, 13, 16]. However, none of these studies focused on certain patient groups or medication classes, like psychiatric drugs.

The aim of this study was to determine the number of ADR reports from Germany in the European ADR database EudraVigilance in which *already known* DDI related to psychiatric drugs and somatic medications were reported to find out whether these still occur in everyday clinical practice. Based on our results, we discuss how to avoid some of these DDI by considering alternative drugs or appropriate measures.

## Material and methods

### Definitions

ADRs (definition is described in literature [17]) can be reported by Health Care Professionals (HCP, e.g. physicians, pharmacists) who are obliged by their professional conduct code to report ADRs or by non-Health Care Professionals (non-HCP, e.g. consumers). A DDI describes a situation in which two drugs affect the activity of each other, i.e. leading to an increased or decreased pharmacological effect of one of these drugs or leading to ADRs that neither or to a lesser extent occur on their own [18]. Thus, efficacy or toxicity (including the ADR profile) of the respective drug(s) may change. DDI can affect pharmacodynamics and pharmacokinetics of these drugs.

### EudraVigilance

EudraVigilance, the ADR database of the European Medicines Agency contains all ADR reports received from the member states of the European Economic Area [19]. In EudraVigilance, ADRs are coded in accordance with the Medical Dictionary for Regulatory Activities (MedDRA) terminology [20] and drugs with the EudraVigilance medicinal product dictionary [21]. In each ADR report, the reporter can assign which drugs are assumed to be suspected, interacting or concomitant [17]. As one of the competent authorities the Federal Institute for Drugs and Medical Devices has access to all data elements of the spontaneous ADR reports including the free-text descriptions (narratives) of the reports.

#### Identification of reports

We identified all spontaneous reports from Germany received between 01/2017 and 12/2021 reported for patients older than 17 years in which antidepressants (anatomical therapeutic classification (ATC) [22] N06A), antipsychotics (ATC N05A) and mood stabilizers (e.g. carbamazepine) were reported as suspected/interacting (*n* = 9,665) (the complete list of drugs analyzed is presented in Additional file 1). We excluded all ADR reports referring to intentional overdoses and suicide attempts (*n* = 9,276 left).

### ABDATA interaction analysis

The ABDATA database is a drug information system of the ABDATA Pharma-Daten-Service (a division of Avoxa—Mediengruppe Deutscher Apotheker GmbH) [23] which was developed primarily for use in pharmacy software as well as in practice management and hospital information systems. The database is updated regularly and supplied as a data package with 5 modules, of which we used the information (i) on active ingredients from the medicinal product module and (ii) on interactions from the drug therapy safety (AMTS, Arzneimitteltherapiesicherheit) module with a version as of 01.01.2022 for our study. Within the AMTS module, each interaction is labeled classifying information concerning its clinical relevance, source of evaluation, data availability, and plausibility of mechanism. In our interaction check, only interactions of pharmacotherapeutics and phytotherapeutics being at least of low clinical relevance were included. The active substances from our reports were linked to the data from the ABDATA database. For each report, all active substances reported as suspected/interacting or concomitant were grouped as pairs, and every possible combination was examined with the interaction table of the AMTS module to detect pDDI.

#### Identification of reports with pDDI according to ABDATA

**Fig. 1:**
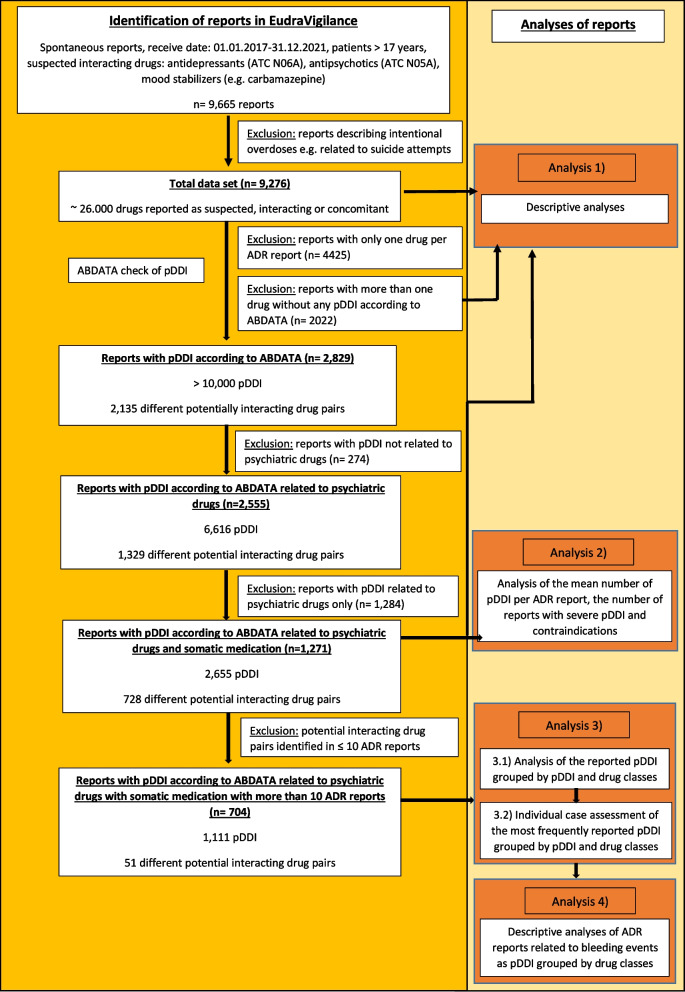
Identification of the ADR reports in EudaVigilance and the pDDI related to psychiatric drugs and somatic medications according to ABDATA. pDDI = potential drug-drug interaction. Figure 1 shows the flowchart for the identification of the ADRs reports in which antidepressants (anatomical therapeutic classification (ATC) [21] N06A), antipsychotics (ATC N05A) and mood stabilizers (e.g. carbamazepine) were reported as suspected/interacting in the European ADR database EudraVigilance and the identification of ADR reports with pDDI related to psychiatric drugs and somatic medications according to ABDATA. The complete list of drugs analyzed is presented in Additional file 1. Further on, the analyses performed are also shown in the flowchart

In the 9,276 reports roughly 26,000 drugs were reported as suspected/interacting or concomitant (Fig. 1). Following application of the ABDATA check, 2,135 different potentially interacting drugs pairs with more than 10,000 pDDI were identified in 2,829 (30.5%) reports. All ADR reports (*n* = 274) containing pDDI not related to psychiatric drugs were excluded and 6,616 pDDI related to 1,329 different potentially interacting drugs pairs including psychiatric drugs in 2,555 (27.5%) reports remained. Thereafter, all potentially interacting drug pairs related to psychiatric drugs only (*n* = 1,284) were excluded. Finally, *n* = 1,271 ADR reports (49.7%) with 728 different potentially interacting drugs pairs related to psychiatric drugs and somatic medications and 2,655 pDDI were identified (Additional file 2). To focus on the most frequently reported pDDI, we excluded all potentially interacting drug pairs with less than 11 reports. Finally, 1,111 pDDI in 704 reports with 51 different potential interacting drug pairs remained.

### Analyses of reports

#### Analysis 1—descriptive analyses

The identified ADR reports related to psychiatric drugs (total data set, *n* = 9,276), the reports with pDDI related to psychiatric drugs and somatic medications according to ABDATA (*n* = 1,271 reports) as well as the reports with more than one drug without any pDDI according to ABDATA were analyzed descriptively (Fig. 1, Analysis 1). In the descriptive analyses the demographical parameters of the patients, the seriousness of the ADR reports, the drugs most frequently reported as suspected/interacting or concomitant, the ADRs most frequently reported, and the primary reporting sources were investigated.

Note that more than one seriousness criterion can be reported per ADR report. The classification of seriousness was performed based on the legal definition and may not correspond to the clinical definition of the severity of an ADR. According to the legal definition, an ADR report is classified as serious, if the ADR was life-threatening, led to hospitalisation or prolongation thereof, death, congenital anomalies or permanent disabilities [17].

The primary reporting source describes the person who reported the ADR. One ADR report can be reported by more than one reporter (e.g. physician and patient independently reported on the same ADR). Our descriptive analysis regarding the primary reporting sources refers to reports which were reported by one reporter only. Notably, reports with more than one reporting source were not excluded from the overall analysis.

#### Analysis 2—analyses of characteristics of reports with pDDI related to psychiatric drugs and somatic medications

In the 1,271 reports with pDDI related to psychiatric drugs and somatic medications according to ABDATA, the number of drugs and pDDI per ADR report as well as the proportions of reports with potentially serious pDDI and contraindications were determined (Fig. 1, Analysis 2). In addition, we analyzed whether the mean number of pDDI and the mean number of drugs reported as suspected/interacting or concomitant increased with patient age. This analysis was also performed for females and males separately (Additional file 3).

#### Analysis 3—analyses of reports with pDDI related to psychiatric drugs and somatic medications with more than 10 ADR reports

The reports with pDDI related to psychiatric drugs and somatic medications with more than 10 ADR reports (*n* = 704 reports, 51 different drug pairs, 1,111 pDDI) (Additional file 2) were grouped by pDDI (= potential pharmacological effects) and the reported drugs according to their superior drug classes (if possible) in order to identify the most frequent pDDI (Fig. 1, Analysis 3.1) (Additional file 4). Note that in one ADR report more than one pDDI could be identified, and thus the number of pDDI per pharmacological effect may exceed the number of reports. In addition, due to the restriction to potentially interacting drug pairs with more than 10 ADR reports, the respective drug classes in our analysis are not a complete representation of these drug classes. An individual case assessment was performed to investigate whether the respective pDDI actually occurred (Fig. 1, Analysis 3.2). Therefore, MedDRA terminology was used to identify the reports with the respective pharmacological effects/ADRs. During the individual case assessment, the causal relationship between the co-exposure of the potentially interacting drug pairs and the occurrence of the ADR were assessed according to WHO criteria [24].

#### Analysis 4—analyses of reports related to bleeding events

In the individual case assessment, bleeding events were identified in a considerable number of reports related to their respective potentially interacting drug pairs. Thus, reports related to bleeding events and reports in which no bleeding events could be identified were analyzed descriptively, too (analyses criteria see above) (Fig. 1, Analysis 4). Additionally, we determined the number of reports in which the respective potentially interacting drug pairs were reported as suspected or interacting.

### Statistical analyses

Means with standard deviations (SD) and medians with interquartile ranges (IQR) were calculated for patients’ age, the number of pDDI and the number of drugs per ADR report. Frequency distributions with percentages were calculated for all other criteria.

#### Calculation of additive risk in the database

Furthermore, we calculated additive relative risks (RR) for the most frequently reported potentially interacting drug pairs and their DDI in the background of the database (detailed description see Additional file 5) [25, 26]. In this calculation the RR of each drug as single effect as well as the relative risk of both drugs as a joined effect are considered. Interaction on an additive scale describes that the joint effect of both drugs is bigger (or smaller) compared to the sum of the single effects of both drugs. For the additive scale the relative excess risk due to interaction (RERI) and the proportion attributable to interaction (AP) which describes the joined effect caused by interaction are shown. If RERI and AP are equal to zero no effect of interaction is assumed, if RERI and AP are > 0 a positive interaction effect (bigger than the sum of the single effects) and if RERI and AP are < 0 a negative interaction effect (smaller than the sum of the single effects) is assumed. As recommended in literature the category with the lowest risk was used as reference. It has to be taken into account that the reference group does not reflect a group of healthy patients not taking any drugs but rather represents patients for whom ADRs related to other drugs than the two drugs examined were reported. Thus, the calculated RR of the respective ADR can be higher for ADR reports not related to the two drugs examined as for the individual drugs or their combination. For this reason, it may be possible that the lowest RR is observed for ADR reports related to one of the individual drugs under consideration which than is used as reference. For a more detailed description for estimating measures of interactions see Knol et al. [25, 26]. Note that the calculation of RR depends on the ADRs reported, thus, reporting biases may influence the calculated RR. Additionally, the calculated RR only applies to the analyzed dataset and may not be transferable to the entire population of patients treated with the respective drugs.

## Results

### Analysis 1—descriptive analyses

The median age of the patients in the total data set (*n* = 9,276) was 49 years and 61.8% (*n* = 5,733) of the patients were females (Table 1). Patients in the reports with pDDI related to psychiatric drugs and somatic medications were older than patients treated with more than one drug without any pDDI (mean 62.3 vs 49.9 years) (t-test *p* < 0.05).

**Table 1:** Analysis 1—descriptive analyses of the total data set, the reports with pDDI related to psychiatric drugs and somatic medications according to ABDATA and the reports with more than one drug reported without any pDDI according to ABDATA

	Total data set (*n* = 9,276)	Reports with pDDI related to psychiatric drugs and somatic medications according to ABDATA (*n* = 1,271)	Reports with more than one drug reported without any pDDI according to ABDATA (*n* = 2,022)
Demographical parameters of the patients			
Mean Age (± SD)	49.9 (± 18.8)	62.3 (± 18.5)	49.2 (± 18.1)
Median Age [± IQR]	49.0 [34–63]	64 [50–78]	49 [36–62]
Female (n, %)	5,733 (61.8%)	803 (63.2%)	1,253 (62.0%)
Male (n, %)	3,478 (37.5%)	462 (36.3%)	754 (37.3%)
Unknown (n, %)	65 (0.7%)	6 (0.5%)	15 (0.7%)
Number of reports per seriousness criterion (%) ^a^			
Serious	3,650 (39.3%)	846 (66.6%)	848 (41.9%)
Death	87 (0.9%)	25 (2.0%)	21 (1.0%)
Life-threatening	267 (2.9%)	73 (5.7%)	59 (2.9%)
Hospitalisation	1,818 (19.6%)	599 (47.1%)	394 (19.5%)
Disabling	100 (1.1%)	16 (1.3%)	32 (1.6%)
The five drugs most frequently reported as suspected, interacting or concomitant (n, %) ^b^			
1	Venlafaxine (1015, 10.9%)	Pantoprazole (362, 28.5%)	Levothyroxine (258, 12.8%)
2	Quetiapine (937, 10.1%)	Acetylsalicylic acid (291, 22.9%)	Quetiapine (238, 11.8%)
3	Mirtazapine (769, 8.3%)	Levothyroxine (256, 20.1%)	Risperidone (230, 11.4%)
4	Risperidone (754, 8.1%)	Torasemide (223, 17.5%)	Aripiprazole (207, 10.2%)
5	Levothyroxine (683, 7.4%)	Metoprolol (220, 17.3%)	Venlafaxine (138, 6.8%)
The five ADRs most frequently reported (n, %) ^c^			
1	Dizziness (765, 8.2%)	Dizziness (127, 10.0%)	Dizziness (156, 7.7%)
2	Nausea (664, 7.2%)	Drug interaction (109, 8.6%)	Fatigue (153, 7.6%)
3	Fatigue (627, 6.8%)	Nausea (97, 7.6%)	Nausea (143, 7.1%)
4	Weight increased (478, 5.2%)	General physical health deterioration (84, 6.6%)	Drug interaction (100, 4.9%)
5	Headache (429, 4.6%)	Fall (83, 6.5%)	Headache (95, 4.7%)
Number of reports per primary source qualification (%) ^d^			
Physician	2,606 (28.1%)	449 (35.3%)	622 (30.8%)
Pharmacist	1,222 (13.2%)	328 (25.8%)	240 (11.9%)
Consumer/non-HCP	4,218 (45.5%)	314 (24.7%)	863 (42.7%)
Other HCP	401 (4.3%)	63 (5.0%)	89 (4.4%)

*pDDI* potential drug-drug interactions

^a^more than one seriousness criterion can be reported per ADR report

^b^drugs can be reported as suspected, interacting or concomitant. More than one drug can be reported per ADR report

^c^more than one ADR can be reported per ADR report

^d^the primary source qualification describes the person who reported the ADR. More than one primary source qualification can be coded in each ADR report (e.g. physician and consumer reporting about the same ADR). Shown is the number of reports containing only one primary source qualification

Considerably more reports related to pDDI of psychiatric drugs and somatic medications were classified as serious (66.6%, *n* = 846) compared to the total data set (39.3%, *n* = 3,650) and to reports with more than one drug reported without any pDDI (41.9%, *n* = 848).

We observed differences regarding the drugs most frequently reported as suspected, interacting or concomitant between the three datasets. Obviously, the five drugs most frequently reported in the reports with pDDI related to psychiatric drugs and somatic medications reflect the most common somatic medications identified as potentially interacting with psychiatric drugs according to ABDATA. These were pantoprazole (362, 28.5%), acetylsalicylic acid (291, 22.9%), levothyroxine (256, 20.1%), torasemide (223, 17.5%) and metoprolol (220, 17.3%).

In the total data set, the ADRs most frequently described were rather unspecific (e.g. dizziness, nausea, fatigue) or well known for the analyzed drugs (e.g. weight increase). Drug interactions were coded in 293 reports (3.2%) of the total data set. Compared to that, a higher proportion of the reports related to pDDI of psychiatric drugs and somatic medications was coded with the term “drug interaction” (8.6%, *n* = 109). Nevertheless, 4.9% (*n* = 100) of the reports with more than one drug without any pDDI according to ABDATA were also coded with this term.

Almost half of the reports of the total data set were created by consumers/non-HCPs (45.5%, *n* = 4,218). In contrast, reports identified with pDDI to psychiatric drugs and somatic medications were more often reported by physicians (35.3%, *n* = 449) and pharmacists (25.8%, *n* = 328) compared to consumers/non-HCP (24.7%, *n* = 314).

### Analysis 2—analyses of characteristics of reports with pDDI related to psychiatric drugs and somatic medications according to ABDATA

In the 1,271 reports, 728 different potentially interacting drug pairs related to psychiatric drugs and somatic medications with in total 2,655 pDDI were found according to ABDATA (Fig. 1, Analysis 2) (list of potentially interacting drug pairs see Additional file 2). In more than half of these reports the pDDI was classified as serious (59.2%, *n* = 753) and in 9.8% (*n* = 125) of the reports the potentially interacting drug pairs were considered contraindicated according to ABDATA (Table 2).

**Table 2:** Analysis 2—analyses of reports with pDDI related to psychiatric drugs and somatic medications according to ABDATA

	Reports with pDDI of potentially interacting drug pairs related to psychiatric drugs and somatic medications according to ABDATA (*n* = 1,271)^a^
Number of reported drugs per ADR report	
Mean (± SD)	7.4 (± 4.3)
Median [± IQR]	7.0 [4–10]
Number of pDDI per ADR report	
Mean (± SD)	2.1 (± 1.8)
Median [± IQR]	1.0 [1.0–2.0]
Number of reports with serious pDDI according to ABDATA	
≥ 1	753 (59.2%)
Number of reports with contraindicated potentially interacting drug pairs related to psychiatric drugs and somatic medication according to ABDATA	
≥ 1	125 (9.8%)
The three most frequently reported contraindicated potentially interacting drug pairs related to psychiatric drugs and somatic medications and their pharmacological effect according to ABDATA	
1	20.8% clozapine and pantoprazole – granulocytopenia/agranulocytosis (26/125)
2	9.6% clozapine and acetylsalicylic acid – granulocytopenia/agranulocytosis (12/125)
3	9.6% clozapine and ramipril – granulocytopenia/agranulocytosis (12/125)

*SD* Standard deviation, *IQR* Interquartile range, *pDDI* potential drug-drug interactions

^a^in the 1,271 reports, 728 potentially interacting drug pairs with 2,655 pDDI were found

**Fig. 2:**
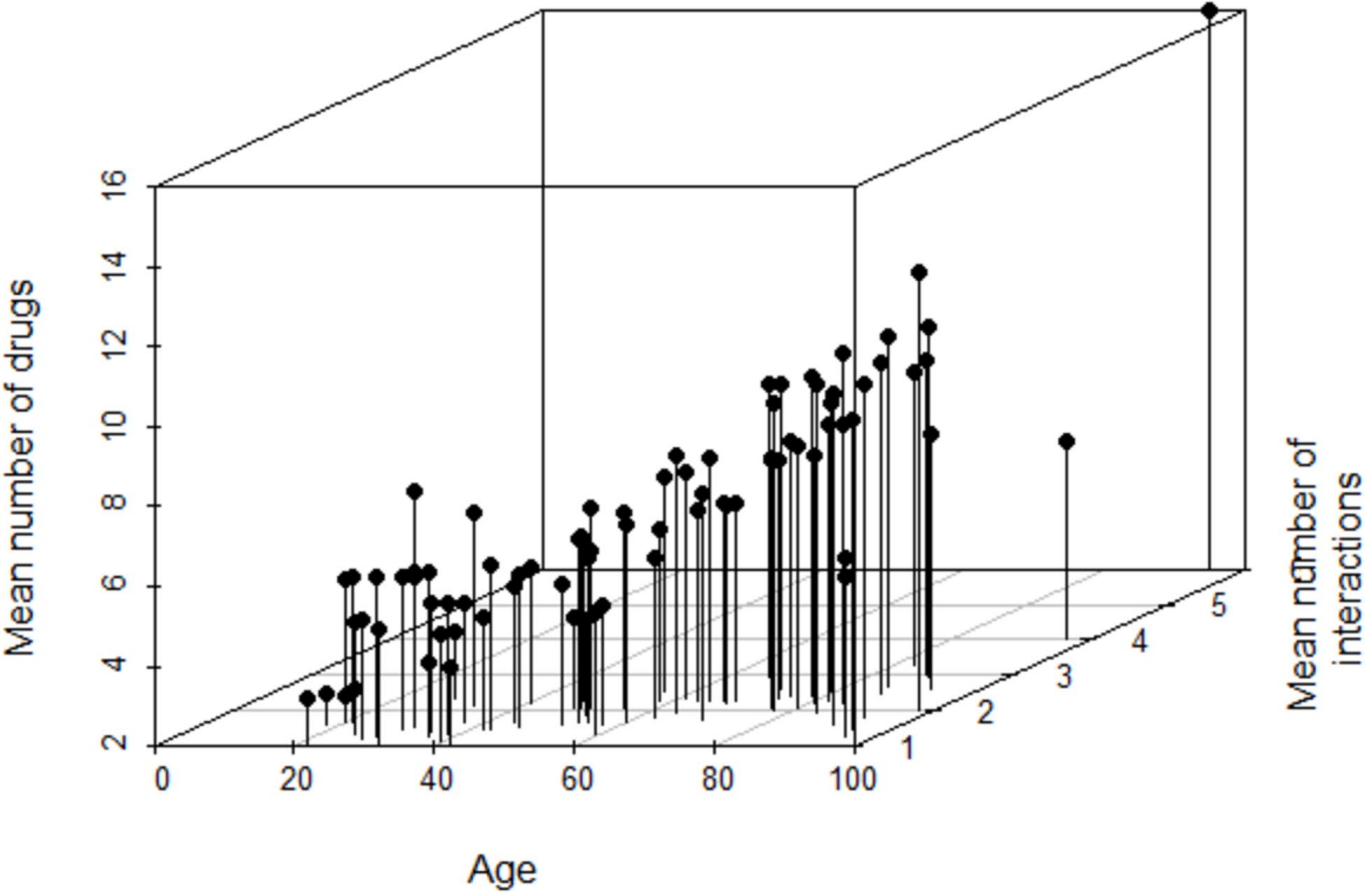
Mean Number of pDDI with psychiatric drugs and somatic medications per ADR report depending on the age of the patients and the mean number of reported drugs per ADR report. Figure 2 shows the mean number of drugs reported as suspected/interacting of concomitants and the mean number of pDDI with psychiatric drugs and somatic medications per ADR report depending on the age of the patients

On average two pDDI and in median one pDDI were identified per report. The mean number of pDDI per ADR report increased with advancing age as well as the mean number of reported drugs per ADR report (Fig. 2). For males, the mean number of pDDI increased up to the age of 75 years and declined afterwards. In contrast, for females a slight increase of the mean number of pDDI was also seen above the age of 80 years. Compared to females younger than 70 years, the mean number of pDDI was partially higher for males of the same age (Additional file 3).

Table 2 shows the number of pDDI per ADR report as well as the number of ADR reports with serious pDDI and pDDI related to contraindicated potentially interacting drugs pairs according to ABDATA.

### Analysis 3—analyses of reports with pDDI related to psychiatric drugs and somatic medications with more than 10 ADR reports

#### Analysis of reported pDDI grouped by pDDI and drug classes

**Fig. 3:**
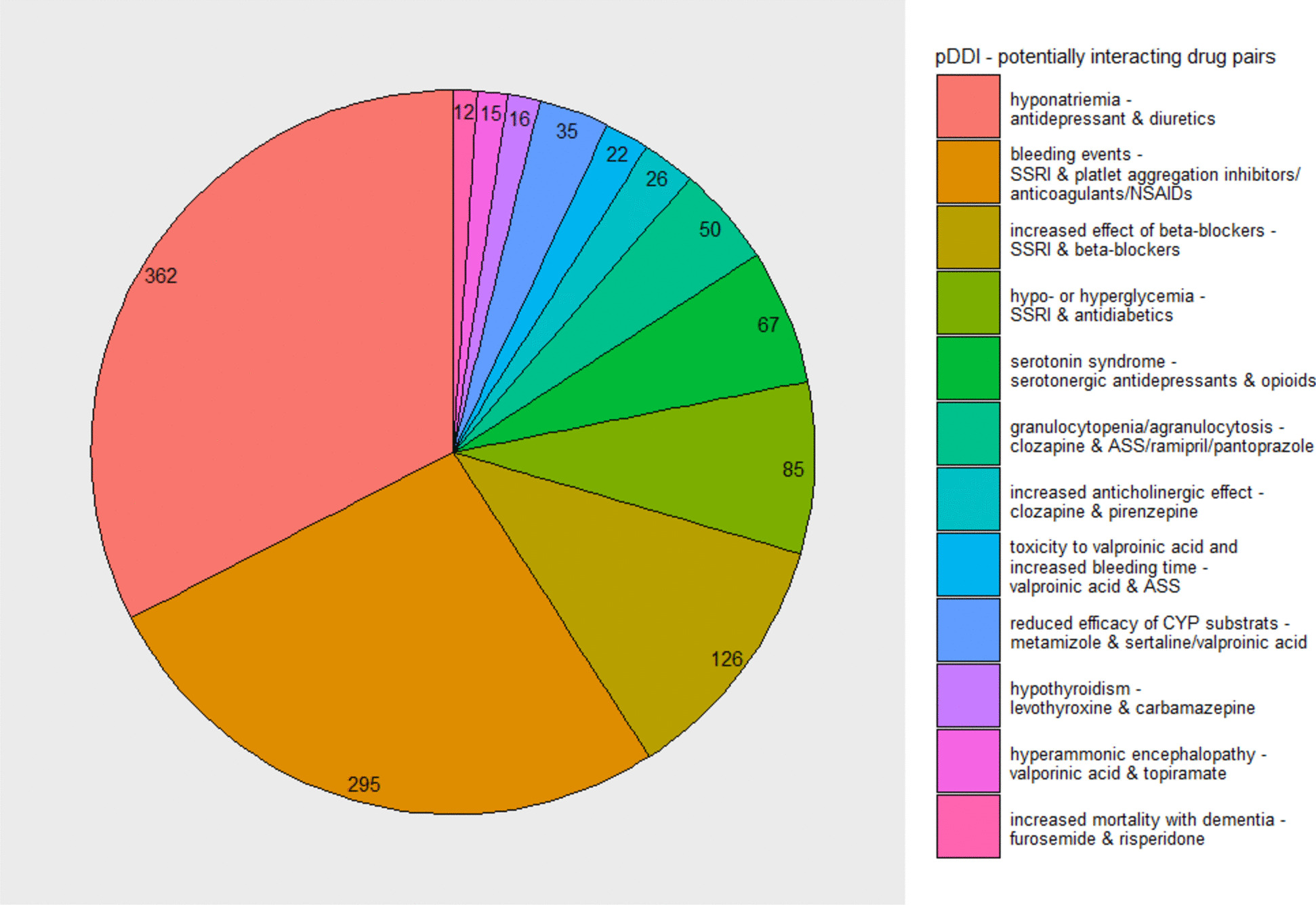
The identified pDDI of the reports of the potentially interacting drug pairs according to ABDATA with more than 10 reports. pDDI = potential drug-drug interactions; SSRI = selective serotonin reuptake inhibitors; NSAIDs = non-steroidal anti-inflammatory drugs; ASS = acetylsalicylic acid; CYP = cytochrome P450. Figure 3 shows the identified 1,111 pDDI (= pharmacological effects) according to ABDATA from the 704 reports of the 51 different potentially interacting drug pairs with more than 10 reports. The pDDI and the potentially interacting drug pairs were grouped by pharmacological effect and drug classes (Additional file 4). Due to the grouping of drug classes, one report could be counted more than once per category. This is the case if more than one potentially interacting drug pair with the same ADR was reported (e.g. bleeding events related to anticoagulants and SSRIs and NSAIDs and SSRIs). Only minor changes in the distribution and the number of reports were seen when the reports were counted once per category

After restricting to the potentially interacting drug pairs with more than 10 reports, 704 reports with 51 different potentially interacting drug pairs describing 1,111 pDDI remained (Fig. 1, Analysis 3.1) (Additional file 4). Grouped by pharmacological effect, the five most frequently identified pDDI were 1) hyponatremias related to antidepressants and diuretics (*n* = 362 pDDI, 32.6% (number of reports *n* = 293)), 2) bleeding events related to SSRIs and platelet aggregation inhibitors, anticoagulants or non-steroidal anti-inflammatory drugs (NSAIDs) (*n* = 295 pDDI, 26.5%, (number of reports *n* = 271)), 3) increased beta-blocker effects (e.g. bradycardia, hypotension) related to SSRIs and beta-blockers (*n* = 126 pDDI, 11.3%, (number of reports *n* = 124)), 4) hypo- or hyperglycemia related to SSRIs and antidiabetics (*n* = 85 pDDI, 7.7%, (number of reports *n* = 77)) and 5) serotonin syndrome related to serotonergic antidepressants and opioids (*n* = 67 pDDI, 6.0%, (number of reports *n* = 58)) (Fig. 3). Considering the ADR reports stratified by reporting source, the most frequently reported pDDI grouped by pharmacological effects and drugs classes in ADR reports from HCP were nearly the same as in the whole dataset, while differences were observed in ADR reports from non-HCP (Additional file 6). For the latter, bleeding events related to SSRIs and platelet aggregation inhibitors, anticoagulants or NSAIDs ranked first (*n* = 66 pDDI, 46.8%, (number of reports *n* = 64)) followed by hyponatremia related to antidepressants and antidiuretics (*n* = 23, 16.3%, (number of reports *n* = 22)).

#### Identification of DDI by individual case assessment in the reports of the most frequently reported potentially interacting psychiatric drugs and somatic medications according to ABDATA

**Fig. 4:**
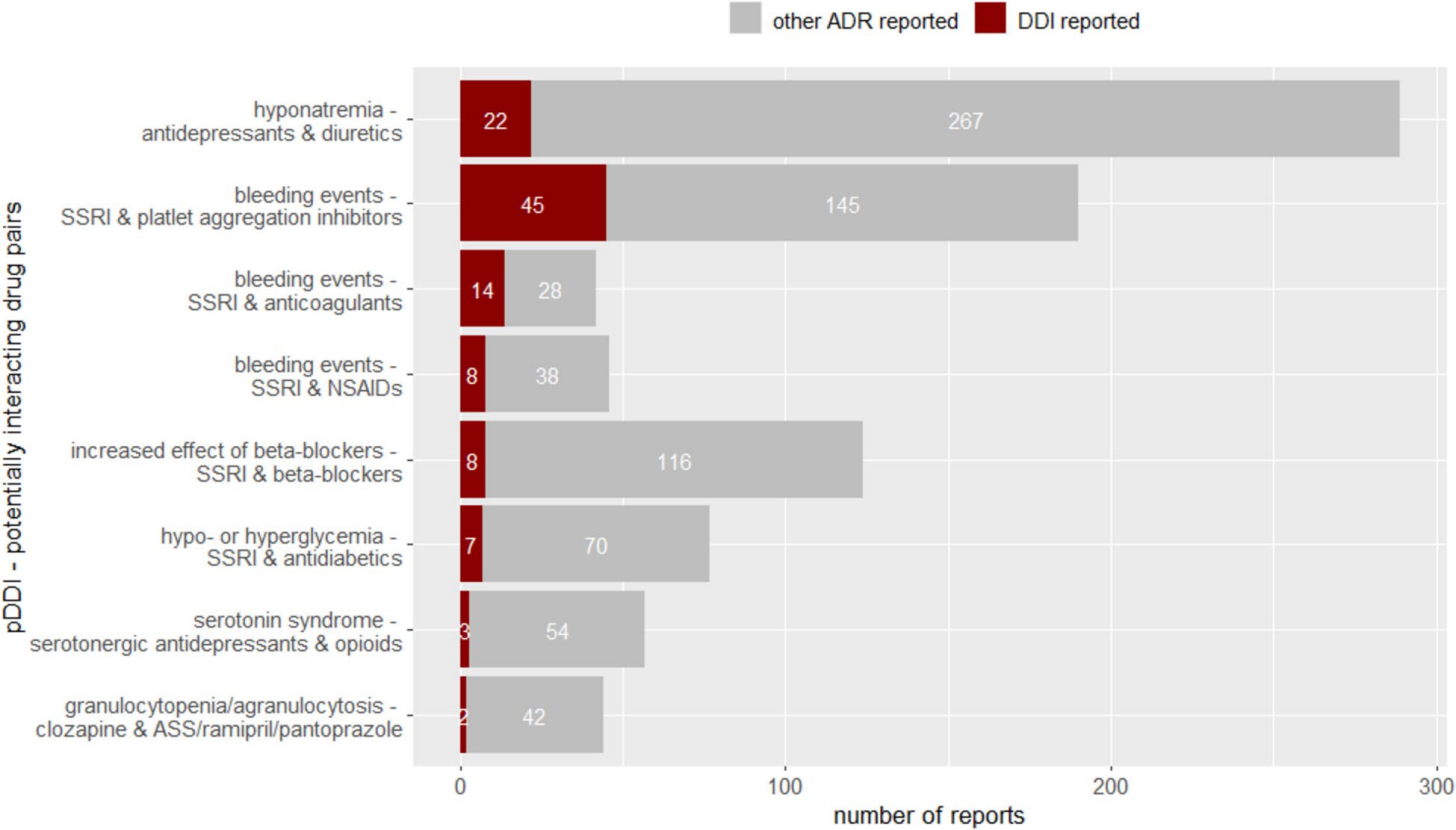
Number of reports describing the specific DDI identified for the potentially interacting drug pairs according to ABDATA. pDDI = potential drug-drug interactions; ADR = adverse drug reactions; SSRI = selective serotonin reuptake inhibitors; NSAIDs = non-steroidal anti-inflammatory drugs. Figure 4 shows the number of reports describing the respective DDI related to the potentially interacting drug pairs according to ABDATA after individual case assessment and the number of reports containing other ADRs

After individual case assessment, bleeding events were found in 33.3% (14/42) of reports related to the potentially interacting drug pairs of SSRIs and anticoagulants (Fig. 1 Analysis 3.2) (Fig. 4). Bleeding events were also reported in 23.7% (45/190) and 17.4% (8/46) of the reports related to the potentially interacting drug pairs of SSRIs and platelet aggregation inhibitors and SSRIs and NSAIDs. Hypo- or hyperglycemia related to SSRIs and antidiabetics were observed in 9.1% (7/77) of the reports. Hyponatremia was reported in 7.6% (22/289) of the reports related to the potentially interacting drug pairs of antidepressants and diuretics.

#### Calculation of additive risk in the database

When considering the relative risk (RR) in the database, the risk to develop the respective pharmacological effect/ADR increased when both drugs of the respective potentially interacting drug pairs were used compared to the use of the single drugs, except for the combination of SSRIs and anticoagulants (Table 3, Additional file 5). In case of the combination of SSRIs and anticoagulants, the relative risk of bleeding events was rather equal for the combination of SSRIs and anticoagulants (RR 19.2 [10.7–34.3]) and anticoagulants alone (RR 19.2 [12.9–28.7]). However, the presence of anticoagulant therapy clearly increased the risk of developing bleeding events in patients treated with SSRIs (RR 19.2 [10.7–34.3]). Note that some of the calculated RR were higher for ADR reports in the absence of both drugs examined because the reference group represents patients taking other drugs than the drugs examined which may also be associated with the respective ADR.

**Table 3:** Calculation of additive risk in the database

Pharmacological effect/ADR – potentially interacting drug pairs^a^					
**Hyponatremia – antidepressants and antidiuretics**^**b**^					
	**Antidepressants absent**		**Antidepressants present**		
	**Hyponatremia reported/hyponatremia not reported**	**RR [95%CI]**	**Hyponatremia reported/hyponatremia not reported**	**RR [95%CI]**	**RR [95% CI] for antidepressants present versus antidepressants absent within strata of antidiuretics**
**Antidiuretics absent**	394/272,482	1 (Reference)	6/3,208	1.3 [0.6–2.9]	1.3 [0.6–2.9]
**Antidiuretics present**	5/719	4.8 [2.0–11.7]	22/267	57.0 [36.5–89.0]	11.9 [4.4–31.6]
**RR [95% CI] for antidiuretics present versus antidiuretics absent within strata of antidepressants**	4.8 [2.0–11.7]		44.1 [17.7–109.6]		
**Measure of interaction on additive scale (RERI) [95% CI]**				51.9 [56.2–77.5]	
**Proportion attributable to interaction (AP) [95% CI]**				0.9 [0.8–1.0]	
**Bleeding events – SSRIs and platelet aggregation inhibitors**^**c**^					
	**SSRIs absent**		**SSRIs present**		
	**Bleeding events reported/bleeding events not reported**	**RR [95%CI]**	**Bleeding events reported/bleeding events not reported**	**RR [95%CI]**	**RR [95% CI] for SSRI absent versus SSRI present within strata of platelet aggregation inhibitors**
**Platelet aggregation inhibitors absent**	17,938/255,387	4.2 [3.1–5.7]	40/2,518	1 (Reference)	4.2 [3.1–5.7]
**Platelet aggregation inhibitors present**	172/858	10.7 [7.6–14.9]	45/145	15.2 [10.2–22.6]	0.7 [0.5–0.9]
**RR [95% CI] for platelet aggregation inhibitors present versus platelet aggregation inhibitors absent within strata of SSRIs**		2.5 [2.2–2.9]		15.2 [10.2–22.6]	
**Measure of interaction on additive scale (RERI) [95% CI]**				−10.3[−18.1-(−2.62)]	
**Proportion attributable to interaction (AP) [95% CI]**				−0.8 [−1.4-(−0.2)]	
**Bleeding events – SSRIs and anticoagulants**^**d**^					
	**SSRIs absent**		**SSRIs present**		
	**Bleeding events reported/bleeding events not reported**	**RR [95%CI]**	**Bleeding events reported/bleeding events not reported**	**RR [95%CI]**	**RR [95% CI] for SSRIs absent versus SSRIs present within strata of anticoagulants**
**Anticoagulants absent**	17,069/255,359	3.6 [2.4–5.4]	24/1,356	1 (Reference)	3.6 [2.4–5.4]
**Anticoagulants present**	1,088/2,165	19.2 [12.9–28.7]	14/28	19.2 [10.7–34.3]	1 [0.7–1.5]
**RR [95% CI] for anticoagulants present versus anticoagulants absent within strata of SSRIs**	5.3 [5.0–5.6]		19.2 [10.7–34.3]		
**Measure of interaction on additive scale (RERI) [95% CI]**				−2.5 [−10.9–5.8]	
**Proportion attributable to interaction (AP) [95% CI]**				−0.1 [−0.6–0.3]	
**Bleeding events – SSRIs and NSAIDs**^**e**^					
	**SSRIs absent**		**SSRIs present**		
	**Bleeding events reported/bleeding events not reported**	**RR [95%CI]**	**Bleeding events reported/bleeding events not reported**	**RR [95%CI]**	**RR [95% CI] for SSRIs absent versus SSRIs present within strata of NSAIDs**
**NSAIDs absent**	18,116/256,094	6.1 [3.8–9.8]	17/1,555	1 (Reference)	6.1 [3.8–9.8]
**NSAIDs present**	54/1,221	3.9 [2.3–6.7]	8/38	16.1 [7.3–35.4]	0.2 [0.1–0.5]
**RR [95% CI] for NSAIDs present versus NSAIDs absent within strata of SSRIs**	0.6 [0.5–0.8]		16.1 [7.3–35.4]		
**Measure of interaction on additive scale (RERI) [95% CI]**				−17.3 [−30.6-(−3.9)]	
**Proportion attributable to interaction (AP) [95% CI]**				−4.4 [−7.4-(−1.5)]	
**Increased beta-blocker effects (hypotension, bradycardia) – SSRIs and beta-blockers**^**f**^					
	**SSRIs absent**		**SSRIs present**		
	**Increased beta-blocker effects reported (e.g. hypotension, bradycardia)/increased beta-blocker effects not reported**	**RR [95%CI]**	**Increased beta-blocker effects reported (e.g. hypotension, bradycardia)/increased beta-blocker effects not reported**	**RR [95%CI]**	**RR [95% CI] for SSRIs absent versus SSRIs present within strata of beta-blockers**
**Beta-blockers absent**	3,688/270,595	3.1 [1.4–6.1]	8/1,816	1 (Reference)	3.1 [1.4–6.1]
**Beta-blockers present**	27/845	7.1 [3.2–15.5]	8/116	14.7 [5.6–38.5]	0.5 [0.2–1.0]
**RR [95% CI] for beta-blockers present versus beta-blockers absent within strata of SSRIs**	2.3 [1.6–3.3]		14.7 [5.6–38.5]		
**Measure of interaction on additive scale (RERI) [95% CI]**				−9.7 [−22.3–2.89]	
**Proportion attributable to interaction (AP) [95% CI]**				−1.4 [−3.0–0.3]	
**Hypo- or hyperglycemia – SSRIs and antidiabetics**^**g**^					
	**SSRIs absent**		**SSRIs present**		
	**Increased beta-blocker effects reported/increased beta-blocker effects not reported**	**RR [95%CI]**	**Increased beta-blocker effects reported/increased beta-blocker effects not reported**	**RR [95%CI]**	**RR [95% CI] for SSRIs absent versus SSRIs present within strata of antidiabetics**
**Antidiabetics absent**	5,720/268,026	10.5 [4.0–28.0]	4/2012	1 (Reference)	10.5 [4.0–28.0]
**Antidiabetics present**	61/1,203	24.3 [8.9–66.6]	7/70	45.8 [13.7–153.0]	0.5 [0.3–1.1]
**RR [95% CI] for antidiabetics present versus antidiabetics absent within strata of SSRIs**	2.3 [1.8–3.0]		45.8 [13.7–153.0]		
**Measure of interaction on additive scale (RERI) [95% CI]**				−31.0 [−76.4–14.4]	
**Proportion attributable to interaction (AP) [95% CI]**				−1.3 [−2.7–0.2]	

*RR* Relative risk, *CI* Confidence intervals, *DDI* Drug-drug interactions

^a^the analysis is based on the drugs of the potentially interacting drug pairs with more than 10 reports. Thus, the drug classes in our analysis are not a complete presentation of these drug classes

^b^the drug class of antidepressants consists of citalopram, escitalopram, duloxetine, mirtazapine, sertraline, venlafaxine, amitriptyline and the drug class of antidiuretics consist of hydrochlorothiazide, furosemide, torasemide and their combination products

^c^the drug class of SSRIs consists of citalopram, sertraline, escitalopram, venlafaxine, duloxetine and the drug class of platelet aggregation inhibitors consists of acetylsalicylic acid and clopidogrel

^d^the drug class of SSRIs consists of citalopram, escitalopram, duloxetine and the drug class of anticoagulants consists of apixaban

^e^the drug class of SSRIs consists of citalopram, venlafaxine, duloxetine and the drug class of NSAIDs consists of ibuprofen

^6^fthe drug class of SSRIs consists of citalopram, duloxetine, sertraline and the drug class of beta-blockers consist of metoprolol and its combination product

^g^the drug class of SSRIs consist of citalopram, duloxetine, venlafaxine, sertraline and the drug class of antidiabetics consists of metformin and sitagliptin

### Analysis 4—analyses of reports related to bleeding events compared to reports without bleeding events related to the respective potentially interacting drug pairs according to ABDATA

Reports in which bleeding events related to SSRIs and platelet aggregation inhibitors, SSRIs and anticoagulants and SSRIs and NSAIDs were found, were in proportion more commonly classified as serious than reports without bleeding events (Fig. 1, Analysis 4) (Table 4). In addition, a higher number of reports were reported by HCP compared to those without bleeding events. In most of the reports describing bleeding events, the respective potentially interacting drug pairs were reported as suspected. However, in only a few reports an interaction was explicitly mentioned by the reporter. Patients with bleeding events related to SSRIs and platelet aggregation inhibitors and SSRIs and anticoagulants were older than those with bleeding events related to SSRIs and NSAIDs. Bleeding events allied with SSRIs and NSAIDs only occurred in females, whereas 48.9% and 42.9% of the bleeding events related to SSRIs and platelet aggregation inhibitors and SSRIs and anticoagulants referred to males. The proportion of males in the reports to SSRIs and platelet aggregation inhibitors and SSRIs and anticoagulants with bleeding events was higher than in those without bleeding events.

**Table 4:** Analysis 4—descriptive analyses of reports with and without bleeding events related to the identified potentially interacting drug pairs according to ABDATA

Potentially interacting drug pair according to ABDATA	Reports on bleeding events	Reports without bleeding events
SSRI and platelet aggregation inhibitors (*n* = 190)		
Number of reports	45 (23.7%)	145 (76.3%)
Age (median [IQR])	75 [65–81]	73 [62–81]
Females	23 (51.1%)	90 (62.1%)
Number of serious reports	42 (93.3%)	104 (71.7%)
Primary reporting source		
Physician	27 (60.0%)	46 (31.7%)
Pharmacist	14 (31.1%)	46 (31.7%)
Other HCP	-	3 (2.1%)
Non-HCP/consumer	1 (2.2%)	42 (29.0%)
Both drugs reported as suspected	42 (93.3%)	19 (13.1%)
Number of reports interaction reported	8 (17.8%)	9 (6.2%)
SSRI and anticoagulants (*n* = 42)		
Number of reports	14 (33.3%)	28 (66.7%)
Age (median [IQR])	75 [72.5–80.8]	76 [69.8–84.2]
Females	8 (57.1%)	19 (67.9%)
Number of serious reports	13 (92.9%)	20 (71.4%)
Primary reporting source		
Physician	9 (64.3%)	9 (32.1%)
Pharmacist	4 (28.6%)	10 (35.7%)
Other HCP	1 (7.1%)	1 (3.6%)
Non-HCP/consumer	-	8 (28.6%)
Both drugs reported as suspected	14 (100.0%)	4 (14.3%)
Number of reports interaction reported	4 (28.6%)	5 (17.8%)
SSRI and NSAIDs (*n* = 46)		
Number of reports	8 (17.4%)	38 (82.6)
Age (median [IQR])	66 [50.2–83.2]	58 [47–68.2]
Females	8 (100.0%)	27 (71.1%)
Number of serious reports	6 (75.0%)	18 (47.4%)
Primary reporting source		
Physician	2 (25.0%)	4 (10.5%)
Pharmacist	5 (62.5%)	7 (18.4%)
Other HCP	-	2 (5.3%)
Non-HCP/consumer	1 (12.5%)	16 (42.1%)
Both drugs reported as suspected	7 (87.5%)	7 (18.4%)
Number of reports interaction reported	1 (12.5%)	3 (7.8%)

*IQR* Interquartile range, *HCP* Health Care Professional, *NSAID* Non-steroidal anti-inflammatory drugs

Table 4 shows the analysis of demographical parameters of the patients, the proportion of reports classified as serious, the number of reports per primary reporting source, as well as the number of reports in which the respective potentially interacting drug pair was reported as suspected/interacting in the reports with and without bleeding events related to SSRIs and platelet aggregation inhibitors, SSRIs and anticoagulants and SSRIs and NSAIDs.

## Discussion

The treatment of patients with severe mental illness and somatic comorbidities is a challenge in everyday clinical practice. In this context, DDI between psychiatric and somatic medications are of great importance. Although these DDIs are generally well-known, they occur frequently due to the complexity of the patients’ conditions and may sometimes even be unavoidable. Nevertheless, DDIs between psychiatric drugs and somatic medications can lead to serious ADRs, thus, alternative treatments should be considered if possible.

Our analysis showed that in roughly 14% of all spontaneous reports from Germany related to psychiatric drugs at least one pDDI with somatic medications could be identified according to the ABDATA database. More than half (58.7%) of these reports contained pDDIs that were classified as serious and roughly a fifth (9.8%) was classified as contraindicated. This proportion illustrates that pDDI are still a relevant problem in the drug treatment process of psychiatric patients.

ADR reports from physicians and pharmacists involved a pDDI more frequently than those from consumers, indicating that physicians and pharmacists are more aware of DDI related ADRs even though only 8.6% of the reports were coded as such according to MedDRA terminology. Additionally, serious ADR were more frequently reported in general by physicians and pharmacists compared to consumers in spontaneous reports from Germany [27].

In our analysis a higher proportion of reports related to psychiatric drugs from consumers could be found, especially in reports where no pDDI was involved. This might reflect the general increase of ADR reports from consumers in EudraVigilance in recent years [28]. Notably, this also applies to the ADR reports referring to psychiatric drugs in our analysis although patients with psychiatric disorders often suffer from listlessness, which may also affect ADR reporting. However, the reports could also have been created by their relatives. In addition, some of the common ADRs related to psychiatric drugs such as weight gain, or other rather subjectively experienced ADRs may affect the quality of life of the patients, which may explain the higher number of reports related to psychiatric drugs from consumers, too [29].

Comparing these reports involving pDDI with reports in which no pDDI was found, remarkable differences in the characteristics of the reports became obvious. Patients in reports involving pDDI were clearly older and a higher proportion of these reports were classified as serious. This was particularly evident for the seriousness criterion hospitalization. Older patients are more prone to DDI as multimorbidity and -as a result- polymedication increases with age [2, 30]. In addition, pharmacokinetics of drugs differ in older adults further increasing the risk for DDIs and ADRs [30]. Finally, DDIs and ADRs in older patients may take a more serious course leading to hospitalization. The same observations were also found in two studies investigating spontaneous reports from Italy without a focus on psychiatric drugs [13, 15]. The slightly higher proportion of pDDI in younger males compared to younger females and older females compared to older males in our study may be related to differences in the occurrence of specific psychiatric diseases and their treatment depending on age and sex [31]. The differences in the distributions could also be related to other reasons or coincidence.

The most frequently detected contraindications in our analysis were clozapine—acetylsalicylic acid, clozapine—pantoprazole and clozapine—ramipril which may lead to drug-induced agranulocytosis, a potentially life-threatening ADR characterized by a decrease in neutrophil count. Hematological monitoring before and during clozapine therapy is mandatory as recorded in the summary of product characteristics [32]. In case of clozapine—acetylsalicylic acid and clozapine—pantoprazole it may also be possible that acetylsalicylic acid and pantoprazole were used as over-the-counter (OTC) drugs by the patients and were not prescribed by physicians. For this reason, patient education and training regarding pDDIs with OTC drugs may be of importance. Furthermore, this also underlines the importance of pharmacists specifically asking patients buying OTC drugs in German pharmacies about previous illnesses and concomitant medications.

Hyponatremia related to antidepressants and diuretics was the most frequently observed pDDI in our analysis. In 9.1% of these reports hyponatremia was reported. Unspecific symptoms, which may be associated with hyponatremia such as altered consciousness, were not considered. The mechanisms leading to hyponatremia are different for both drug classes, but their combination results in an even higher risk of developing hyponatremia [33, 34]. Since hyponatremia can take a severe course e.g. coma, we would like to emphasize the importance of the regular monitoring of sodium levels if patients are treated with antidepressants and diuretics as specified in the summary of product characteristics (SmPC). Older patients, patients additionally treated with K + -sparing diuretics such as spironolactone, patients with malignancies, pulmonary and kidney diseases as well as females may carry a higher risk to develop hyponatremia [33–35]. In general, beta-blockers and calcium antagonists or non-serotonergic antidepressants [34, 35] may be considered as therapeutic alternatives to treat hypertension or depression.

Bleeding events related to SSRIs and platelet aggregation inhibitors, anticoagulants and NSAIDs were the second most frequently identified pDDI in our analysis. In one third (33.3%) of the reports related to SSRIs and anticoagulants, 23.7% of reports related to SSRIs and platelet aggregation inhibitors and 17.4% of reports related to SSRIs and NSAIDs, the bleeding events could be confirmed. Bleeding events related to SSRIs and anticoagulants were also found as one of the most frequent drug interactions in a study of spontaneous reports from Italy [16]. SSRIs reduce the ability of platelets to aggregate thereby increasing the risk of bleeding, especially in combination with other drugs affecting blood clotting [36, 37]. Further on, SSRIs may increase gastric acid secretion thereby increasing the vagal tone subsequently leading to potential ulcerogenic effects and gastrointestinal bleeding [36–38]. The current guideline for the treatment of coronary heart diseases and myocardial infarctions suggests the concomitant use of SSRI and anticoagulant drugs [39], which may explain the frequent presence of this combination in our analysis. As an alternative switching to non-serotonergic antidepressants may be preferable [40], especially for patients who carry a higher risk of bleeding events such as patients with bleeding events in the past. Regular monitoring for bleeding events of patients using SSRIs and other drugs affecting coagulation may be considered. Regarding NSAIDs the inhibition of cyclooxygenase (COX-) 1 and 2 impacts on the gastric mucosal barrier and may favor ulcer formation and thereby upper gastrointestinal bleeding [41]. If the combination of NSAIDs and SSRIs cannot be avoided, protective agents such as proton-pump inhibitors may be prescribed additionally to reduce the risk of gastrointestinal bleedings. Anyway, bleeding events may be more commonly reported in our analysis since these often require medical treatment and may be easier to recognize.

The third most common pDDI was increased effects of beta-blockers related to the combination of beta-blockers and SSRIs. In 6.9% of these reports, bradycardia and hypotension were reported. Rather unspecific symptoms such as dizziness or falls, which could also be symptoms of hypotension or bradycardia, were not considered. The increased effect of beta-blockers which are substrates of CYP2D6 such as metoprolol results from the inhibition of CYP2D6 by SSRIs leading to increased plasma concentrations of these beta-blockers, thereby inducing hypotension, bradycardia, and consecutively falls [42]. SSRIs such as fluoxetine or paroxetine are potent inhibitors and SSRIs like citalopram or escitalopram are weak inhibitors of CYP2D6. Obviously, the risk of increased beta-blocker effects is expected to be higher with SSRIs which are potent CYP2D6 inhibitors, as also observed by others [42, 43]. However, in our analysis, bradycardia and hypotension were also reported for the concomitant use of SSRIs, which are weak inhibitors of CYP2D6. Thus, prescribing beta-blockers not metabolized by CYP2D6 such as bisoprolol [44] or a careful monitoring of beta-blocker plasma concentration in patients treated with both drug classes and consequent dose adjustments may be recommended [43, 44] if the combination cannot be avoided.

Hypo- or hyperglycemic events related to SSRIs and antidiabetics was the fourth common identified pDDI in our analysis. In 9.0% of these reports hypo- or hyperglycemic events were reported. However, symptoms possibly associated with hypo- and hyperglycemic events were not taken into account. Literature is inconsistent, for some SSRIs improved and for others worsened blood glucose control was reported [45–48]. In addition, depression itself may interfere with blood glucose control, too. More recently, the role of metformin to act as an antidepressant in patients with diabetes was studied [49]. Metformin leads to a decrease of chronic inflammation in diabetes patients by several pathways and, thereby, a decrease of symptoms of depression. Further research is needed to clarify the observations. Anyway, a regular monitoring of blood glucose concentrations by the patients as well as adjusting the dosages of insulin or antidiabetic drugs may be necessary.

In only 5.3% of reports related to serotonergic antidepressants and serotonergic opioids, serotonin syndrome was described. In brevity, serotonergic antidepressants increase the synaptic concentration of serotonin [50]. Serotonergic opioids inhibit the reuptake of serotonin by inhibiting the serotonin transporter, leading to an increase of synaptic serotonin concentrations, too. Non-serotonergic antidepressants may be used as an alternative treatment.

### Strengths and limitations

One of the strengths of analyses performed in spontaneous reporting databases is the inclusion of a diverse study population without any predefined in- and exclusion criteria like in clinical trials [29]. Hence, patients with comorbidities taking multiple drugs, or in case of this analysis patients with severe mental illnesses, are also included. An additional strength of this analysis is, that an individual assessment of reports was performed evaluating the co-exposure and the causal relationship between the intake of the respective drugs and the occurrence of the ADR/s in the reports with the most frequently reported pDDIs.

One of the major limitations of the analyses of spontaneous reports is the unknown amount of underreporting [51, 52] which may also differ for specific drugs and serious, non-serious or unexpected ADRs [53]. For this reason, the calculated RR have to be interpreted with caution as the calculation of RR depends on the ADRs reported and, thus, may also be influenced by reporting biases. In addition, the number of drug-exposed patients not experiencing an ADR is unknown. Thus, incidences cannot be calculated based on spontaneous report analyses [29].

Besides underreporting, the quality of documentation may differ between the reports. Especially information regarding concomitant drugs or medical histories of the patients may not be (fully) reported. In case of concomitant drugs, this would influence our analysis, as we have created a list of all possible drug-drug combinations per ADR report based on all reported drugs. With respect to the patient's medical history, we were unable to perform an analysis of associated factors such as comorbidities because the number of reports with confirmed interactions and reported patients’ histories was too low.

We only used one database for identification of DDI. The results of other databases which could also be used for the detection of DDI may differ as observed by others [54, 55]. Further on, the sensitivity, specificity and accuracy to detect DDI in eight databases (including ABDATA) commonly used in the German healthcare system differed [56]. Thus, we cannot rule out that some interactions remained undetected because they were not included in the ABDATA database. Further on, we only detected reports with already known pDDIs. Thus, the results of our study may not apply to unknown or new DDIs, which were not the subject of this analysis. In addition, the dosage of the respective drugs was not considered, which may have already been adjusted to avoid some of the DDI.

## Conclusion

Our analysis shows that well-known DDI still occur to a considerable amount in the treatment of psychiatric patients with psychiatric drugs and somatic medication. Whenever possible, other drug combinations with a lower potential of DDIs or appropriate monitoring measures may be considered. Special attention should be paid to older patients receiving polymedication. Further on, patients should be informed about pDDI, especially regarding OTC drugs.

## Supplementary Information

Supplementary Material 1.

Supplementary Material 2.

Supplementary Material 3.

Supplementary Material 4.

Supplementary Material 5.

Supplementary Material 6.

## Data Availability

The pseudonymised ADR reports from EudraVigilance are not publicly available and cannot be provided upon request due to data protection requirements. In order to fulfill their legal obligations distinct levels of access to EudraVigilance are provided for various stakeholders ([https://www.ema.europa.eu/en/human-regulatory/research-development/pharmacovigilance/eudravigilance/access-eudravigilance-data](https:/www.ema.europa.eu/en/human-regulatory/research-development/pharmacovigilance/eudravigilance/access-eudravigilance-data)). Being one of the competent authorities in Germany, the Federal Institute for Drugs and Medical Devices is granted with the highest level of access covering the individual spontaneous adverse drug reaction (ADR) reports. This access to the individual spontaneous ADR reports from EudraVigilance is not granted to the public and cannot be provided upon request due to data protection requirements. However, a lower level of access is granted to the public thereby enabling researchers to perform the same analysis in EudraVigilance albeit with aggregated data (public access: [www.adrreports.eu/en/index.html](http:/www.adrreports.eu/en/index.html)). For further information regarding the processing of personal data in the context of the operation of EudraVigilance Human we refer to the European Medicines Agency’s Data Protection Notice for EudraVigilance Human.

## Abbreviations

ADR: Adverse drug reaction
AMTS: “Arzneimitteltherapiesicherheit”
ATC: Anatomical therapeutic classification
CI: Confidence intervals
DDI: Drug-drug interactions
HCP: Health Care Professional
IQR: Interquartile range
Non-HCP: Non-Health Care Professional
NSAID: Non-steroidal anti-inflammatory drugs
MedDRA: Medical Dictionary for Regulatory Activities
OTC drugs: Over-the-counter drugs
pDDI: Potential drug-drug interactions
SD: Standard deviation
SMI: Severe mental illness
SmPC: Summary of product characteristics
SSRI: Selective serotonin reuptake inhibitors
RR: Relative risk
