# Supplementary material for: Analysis of drug-drug interactions in spontaneous adverse drug reaction reports from EudraVigilance focusing on psychiatric drugs and somatic medication
Source: BMC Psychiatry. 2025 Oct 2;25:914. doi: 10.1186/s12888-025-07352-8 (PMC12490046; doi:10.1186/s12888-025-07352-8)
Supplement: Supplementary file 1 — Supplementary Material 1. [file 12888_2025_7352_MOESM1_ESM.pdf]

Additional file 1) List of drugs for identification of psychiatric drugs.

**Drugs**	**ATC code**
Levomepromazine	N05AA02
Fluphenazine	N05AB02
Perphenazine	N05AB03
Perazine	N05AB10
Thioridazine	N05AC02
Haloperidol	N05AD01
Melperone	N05AD03
Pipamperone	N05AD05
Bromperidol	N05AD06
Benperidol	N05AD07
Sterindole	N05AE03
Ziprasidone	N05AE04
Lurasidone	N05AE05
Flupentixol	N05AF01
Chlorprothixene	N05AF03
Zuclopenthixol	N05AF05
Fluspirilene	N05AG01
Pimozide	N05AG02
Loxapine	N05AH01
Clozapine	N05AH02
Olanzapine	N05AH03
Quetiapine	N05AH04
Asenapine	N05AH05
Sulpiride	N05AL01
Tiapride	N05AL03
Amisulpride	N05AL05
Lithium	
Prothipendyl	N05AX07
Risperidone	N05AX08
Aripiprazole	N05AX12
Dehydroaripiprazole	N05AX12
Paliperidone	N05AX13
Cariprazine	N05AX15
Brexpiprazole	N05AX16
Imipramine	N06AA02
Trimipramine	N06AA06
Imipraminoxide	N06AA03
Clomipramine	N06AA04
Desmethylclomipramine	N06AA04
Opipramol	N06AA05
Amitriptyline	N06AA09
Nortriptyline	N06AA10
Desmethyldoxepin	N06AA12
Doxepin	N06AA12
Dosulepin	N06AA16
Maprotiline	N06AA21
Fluoxetine	N06AB03

Norfluoxetine	N06AB03
Citalopram	N06AB04
Desmethylcitalopram	N06AB04
Escitalopram	N06AB10
Paroxetine	N06AB05
Desmethylsertraline	N06AB06
Sertraline	N06AB06
Fluvoxamine	N06AB08
Tranylcypromine	N06AF04
Moclobemide	N06AG02
Mianserin	N06AX03
Trazodone	N06AX05
Mirtazapine	N06AX11
Tianeptine	N06AX14
Venlafaxine	N06AX16
Milnacipran	N06AX07
Duloxetine	N06AX21
Bupropion	N06AX12
Agomelatine	N06AX22
Carbamazepine	N03AF01
Oxcarbamazepine	N03AF02
Valproinic acid	N03AG01
Lamotrigine	N03AX09
