# Supplementary material for: Analysis of drug-drug interactions in spontaneous adverse drug reaction reports from EudraVigilance focusing on psychiatric drugs and somatic medication
Source: BMC Psychiatry. 2025 Oct 2;25:914. doi: 10.1186/s12888-025-07352-8 (PMC12490046; doi:10.1186/s12888-025-07352-8)
Supplement: Supplementary file 2 — Supplementary Material 2. [file 12888_2025_7352_MOESM2_ESM.pdf]

Additional file 2) Identified potentially interacting drug pairs of psychiatric drugs and somatic medications.

**Drug 1**	**ATC code Drug 1**	**Drug 2**	**ATC code Drug 2**	**Number of reports**
Mirtazapine	N06AX11	Torasemide	C03CA04	71
Acetylsalicylic acid	A01AD05	Citalopram	N06AB04	55
Metoprolol	C07AB02	Citalopram	N06AB04	45
Acetylsalicylic acid	A01AD05	Duloxetine	N06AX21	41
Acetylsalicylic acid	A01AD05	Venlafaxine	N06AX16	40
Mirtazapine	N06AX11	Duloxetine	N06AX21	39
Citalopram	N06AB04	Torasemide	C03CA04	37
Mirtazapine	N06AX11	Hydrochlorothiazide	C03AA03	33
Metoprolol	C07AB02	Duloxetine	N06AX21	33
Acetylsalicylic acid	A01AD05	Escitalopram	N06AB10	30
Duloxetine	N06AX21	Torasemide	C03CA04	28
Acetylsalicylic acid	A01AD05	Sertraline	N06AB06	28
Metoprolol	C07AB02	Escitalopram	N06AB10	27
Clozapine	N05AH02	Pantoprazole	A02BC02	26
Clozapine	N05AH02	Pirenzepine	A02BX03	26
Venlafaxine	N06AX16	Torasemide	C03CA04	25
Sertraline	N06AB06	Torasemide	C03CA04	24
Metformin	A10BA02	Venlafaxine	N06AX16	24
Metformin	A10BA02	Duloxetine	N06AX21	23
Valproic acid	N03AG01	Acetylsalicylic acid	A01AD05	22
Citalopram	N06AB04	Hydrochlorothiazide	C03AA03	22
Venlafaxine	N06AX16	Hydrochlorothiazide	C03AA03	22
Metoprolol	C07AB02	Sertraline	N06AB06	21
Apixaban	B01AF02	Citalopram	N06AB04	20
Duloxetine	N06AX21	Hydrochlorothiazide	C03AA03	19
Sertraline	N06AB06	Metamizole	M01BA07	18
Duloxetine	N06AX21	Tilidine	N02AX01	17
Valproic acid	N03AG01	Metamizole	M01BA07	17
Escitalopram	N06AB10	Torasemide	C03CA04	17
Risperidone	N05AX08	Duloxetine	N06AX21	16
Amitriptyline	N06AA09	Duloxetine	N06AX21	16
Escitalopram	N06AB10	Hydrochlorothiazide	C03AA03	16
Ibuprofen	C01EB16	Duloxetine	N06AX21	16
Levothyroxine	H03AA51	Carbamazepine	N03AF01	16
Sertraline	N06AB06	Hydrochlorothiazide	C03AA03	15
Valproic acid	N03AG01	Topiramate	N02CX12	15
Ibuprofen	C01EB16	Venlafaxine	N06AX16	15
Ibuprofen	C01EB16	Citalopram	N06AB04	15
Metformin	A10BA02	Citalopram	N06AB04	15
Venlafaxine	N06AX16	Tilidine	N02AX01	13
Oxycodone	N02AA05	Amitriptyline	N06AA09	13
Clozapine	N05AH02	Acetylsalicylic acid	A01AD05	12
Clozapine	N05AH02	Ramipril	C09AA05	12
Citalopram	N06AB04	Oxycodone	N02AA05	12
Duloxetine	N06AX21	Oxycodone	N02AA05	12

Risperidone	N05AX08	Furosemide	C03CA01	12
Apixaban	B01AF02	Duloxetine	N06AX21	12
Clopidogrel	B01AC04	Citalopram	N06AB04	12
Metformin	A10BA02	Sertraline	N06AB06	12
Amitriptyline	N06AA09	Torasemide	C03CA04	11
Citalopram	N06AB04	Furosemide	C03CA01	11
Mirtazapine	N06AX11	Furosemide	C03CA01	11
Apixaban	B01AF02	Escitalopram	N06AB10	11
Sitagliptin	A10BD24	Duloxetine	N06AX21	11
Potential interacting drug pairs with < 11 ADR reports				
Citalopram	N06AB04	Omeprazole	A02BC01	10
Mirtazapine	N06AX11	Xipamide	C03BA10	10
Ibuprofen	C01EB16	Escitalopram	N06AB10	10
Metformin	A10BA02	Escitalopram	N06AB10	10
Tramadol	N02AJ13	Duloxetine	N06AX21	10
Tilidine	N02AX01	Amitriptyline	N06AA09	10
Tramadol	N02AJ13	Mirtazapine	N06AX11	10
Opipramol	N06AA05	Hydrochlorothiazide	C03AA03	9
Amitriptyline	N06AA09	Hydrochlorothiazide	C03AA03	9
Citalopram	N06AB04	Fentanyl	N01AH01	9
Opipramol	N06AA05	Torasemide	C03CA04	9
Duloxetine	N06AX21	Tapentadol	N02AX06	9
Sitagliptin	A10BD24	Venlafaxine	N06AX16	9
Phenprocoumon	B01AA04	Duloxetine	N06AX21	9
Apixaban	B01AF02	Sertraline	N06AB06	9
Doxepine	D04AX01	Sertraline	N06AB06	9
Metformin	A10BA02	Lamotrigine	N03AX09	9
Metoprolol	C07AB02	Bupropion	N06AX12	9
Lamotrigine	N03AX09	Ethinylestradiol	G03CA01	8
Citalopram	N06AB04	Tilidine	N02AX01	8
Bupropion	N06AX12	Metamizole	M01BA07	8
Duloxetine	N06AX21	Buprenorphine	N02AE01	8
Phenprocoumon	B01AA04	Escitalopram	N06AB10	8
Rivaroxaban	B01AF01	Escitalopram	N06AB10	8
Apixaban	B01AF02	Venlafaxine	N06AX16	8
Rivaroxaban	B01AF01	Duloxetine	N06AX21	8
Doxepine	D04AX01	Citalopram	N06AB04	8
Tramadol	N02AJ13	Citalopram	N06AB04	8
Acetylsalicylic acid	A01AD05	Milnacipran	N06AX17	8
Tramadol	N02AJ13	Amitriptyline	N06AA09	8
Amlodipine	C08CA01	Carbamazepine	N03AF01	8
Simvastatin	C10AA01	Carbamazepine	N03AF01	8
Doxepine	D04AX01	Quetiapine	N05AH04	8
Doxepine	D04AX01	Risperidone	N05AX08	8
Haloperidol	N05AD01	Pirenzepine	A02BX03	7
Amisulpride	N05AL05	Pirenzepine	A02BX03	7
Risperidone	N05AX08	Metoclopramide	A03FA01	7
Citalopram	N06AB04	Metoclopramide	A03FA01	7
Sertraline	N06AB06	Oxycodone	N02AA05	7

Venlafaxine	N06AX16	Oxycodone	N02AA05	7
Duloxetine	N06AX21	Fentanyl	N01AH01	7
Doxepine	N06AA12	Torasemide	C03CA04	7
Clopidogrel	B01AC04	Venlafaxine	N06AX16	7
Sitagliptin	A10BD24	Citalopram	N06AB04	7
Metformin	A10BA02	Paroxetine	N06AB05	7
Duloxetine	N06AX21	Trazodone	N06AX05	7
Apixaban	B01AF02	Carbamazepine	N03AF01	7
Hydrochlorothiazide	C03AA03	Carbamazepine	N03AF01	7
Opipramol	N06AA05	Duloxetine	N06AX21	6
TrImipramine	N06AA06	Duloxetine	N06AX21	6
Doxepine	N06AA12	Duloxetine	N06AX21	6
Trazodone	N06AX05	Hydrochlorothiazide	C03AA03	6
Duloxetine	N06AX21	Metoclopramide	A03FA01	6
Mirtazapine	N06AX11	Dimenhydrinate	A04AB02	6
Sertraline	N06AB06	Fentanyl	N01AH01	6
Lamotrigine	N03AX09	Dienogest	G03AA16	6
Tramadol	N02AJ13	Escitalopram	N06AB10	6
Doxepine	D04AX01	Venlafaxine	N06AX16	6
Duloxetine	N06AX21	Venlafaxine	N06AX16	6
Carvedilol	C07AG02	Duloxetine	N06AX21	6
Enoxaparin	B01AB05	Citalopram	N06AB04	6
Rivaroxaban	B01AF01	Citalopram	N06AB04	6
Duloxetine	N06AX21	Citalopram	N06AB04	6
Sitagliptin	A10BD24	Sertraline	N06AB06	6
Edoxaban	B01AF03	Sertraline	N06AB06	6
Pantoprazole	A02BC02	Fluoxetine	N06AB03	6
Tramadol	N02AJ13	Carbamazepine	N03AF01	6
Tramadol	N02AJ13	Quetiapine	N05AH04	6
Lithium	N05AN01	Ramipril	C09AA05	6
Doxepine	N06AA12	Hydrochlorothiazide	C03AA03	5
Pipamperone	N05AD05	Pirenzepine	A02BX03	5
Citalopram	N06AB04	Xipamide	C03BA10	5
Sertraline	N06AB06	Tilidine	N02AX01	5
Sertraline	N06AB06	Levofloxacin	J01MA12	5
Risperidone	N05AX08	Solifenacin	G04BD08	5
Clopidogrel	B01AC04	Escitalopram	N06AB10	5
Diclofenac	D11AX18	Escitalopram	N06AB10	5
Diclofenac	D11AX18	Venlafaxine	N06AX16	5
Tizanidine	M03BX02	Venlafaxine	N06AX16	5
Clopidogrel	B01AC04	Duloxetine	N06AX21	5
Phenprocoumon	B01AA04	Citalopram	N06AB04	5
Nebivolol	C07AB12	Citalopram	N06AB04	5
Ibuprofen	C01EB16	Sertraline	N06AB06	5
Phenprocoumon	B01AA04	Sertraline	N06AB06	5
Clopidogrel	B01AC04	Sertraline	N06AB06	5
Ibuprofen	C01EB16	Milnacipran	N06AX17	5
Duloxetine	N06AX21	Milnacipran	N06AX17	5
Acetylsalicylic acid	A01AD05	Fluoxetine	N06AB03	5

Metoprolol	C07AB02	Paroxetine	N06AB05	5
Tamsulosin	G04CA02	Bupropion	N06AX12	5
Furosemide	C03CA01	Carbamazepine	N03AF01	5
Atorvastatin	C10AA05	Carbamazepine	N03AF01	5
Topiramate	N02CX12	Carbamazepine	N03AF01	5
Doxepine	D04AX01	Pipamperone	N05AD05	5
Lithium	N05AN01	Duloxetine	N06AX21	5
Lithium	N05AN01	Enalapril	C09AA02	5
Escitalopram	N06AB10	Omeprazole	A02BC01	4
Haloperidol	N05AD01	Metoclopramide	A03FA01	4
Melperon	N05AD03	Metoclopramide	A03FA01	4
Escitalopram	N06AB10	Metoclopramide	A03FA01	4
Mirtazapine	N06AX11	Chlortalidone	C03BA04	4
Clozapine	N05AH02	Ibuprofen	C01EB16	4
Escitalopram	N06AB10	Tilidine	N02AX01	4
Lamotrigine	N03AX09	Paracetamol	N02BE01	4
Duloxetine	N06AX21	Furosemide	C03CA01	4
Venlafaxine	N06AX16	Tapentadol	N02AX06	4
Mirtazapine	N06AX11	Cetirizine	R06AE07	4
Clozapine	N05AH02	Carbidopa	NN04BA02N	4
Sitagliptin	A10BD24	Escitalopram	N06AB10	4
Enoxaparin	B01AB05	Escitalopram	N06AB10	4
Carvedilol	C07AG02	Escitalopram	N06AB10	4
Duloxetine	N06AX21	Escitalopram	N06AB10	4
Rivaroxaban	B01AF01	Venlafaxine	N06AX16	4
Domperidone	A03FA03	Venlafaxine	N06AX16	4
Tramadol	N02AJ13	Venlafaxine	N06AX16	4
Propranolol	C07AA05	Duloxetine	N06AX21	4
Nebivolol	C07AB12	Duloxetine	N06AX21	4
Diclofenac	D11AX18	Duloxetine	N06AX21	4
Etoricoxib	M01AH05	Duloxetine	N06AX21	4
Nebivolol	C07AB12	Sertraline	N06AB06	4
Ibuprofen	C01EB16	Fluoxetine	N06AB03	4
Metoprolol	C07AB02	Fluoxetine	N06AB03	4
Duloxetine	N06AX21	Fluoxetine	N06AB03	4
Phenprocoumon	B01AA04	Opipramol	N06AA05	4
Phenprocoumon	B01AA04	Amitriptyline	N06AA09	4
Fentanyl	N01AH01	Amitriptyline	N06AA09	4
Oxycodone	N02AA05	Doxepine	N06AA12	4
Tramadol	N02AJ13	Doxepine	N06AA12	4
Tilidine	N02AX01	Doxepine	N06AA12	4
Torasemide	C03CA04	Carbamazepine	N03AF01	4
Prednisolone	A01AC04	Carbamazepine	N03AF01	4
Doxepine	D04AX01	Olanzapine	N05AH03	4
Levomethadone	N02AC06	Olanzapine	N05AH03	4
Amiodarone	C01BD01	Risperidone	N05AX08	4
Doxepine	D04AX01	Melperon	N05AD03	4
Tramadol	N02AJ13	Melperon	N05AD03	4
Moxonidine	C02AC05	Mirtazapine	N06AX11	4

Tilidine	N02AX01	TrImipramine	N06AA06	4
Clozapine	N05AH02	Hydrochlorothiazide	C03AA03	3
Imipramine	N06AA02	Hydrochlorothiazide	C03AA03	3
Paroxetine	N06AB05	Hydrochlorothiazide	C03AA03	3
Milnacipran	N06AX17	Hydrochlorothiazide	C03AA03	3
Paroxetine	N06AB05	Pantoprazole	A02BC02	3
Escitalopram	N06AB10	Esomeprazole	A02BC05	3
Zuclopenthixol	N05AF05	Pirenzepine	A02BX03	3
Risperidone	N05AX08	Pirenzepine	A02BX03	3
Duloxetine	N06AX21	Xipamide	C03BA10	3
Sertraline	N06AB06	Chlortalidone	C03BA04	3
Valproic acid	N03AG01	Ethinylestradiol	G03CA01	3
Agomelatine	N06AX22	Ethinylestradiol	G03CA01	3
Citalopram	N06AB04	Amiodarone	C01BD01	3
Opipramol	N06AA05	Celecoxib	L01XX33	3
Venlafaxine	N06AX16	Fentanyl	N01AH01	3
Citalopram	N06AB04	Buprenorphine	N02AE01	3
TrImipramine	N06AA06	Torasemide	C03CA04	3
Paroxetine	N06AB05	Torasemide	C03CA04	3
Milnacipran	N06AX17	Torasemide	C03CA04	3
Doxepine	N06AA12	Furosemide	C03CA01	3
Quetiapine	N05AH04	Ethanol	B05XX04	3
Escitalopram	N06AB10	Linezolid	J01XX08	3
Pipamperone	N05AD05	Solifenacin	G04BD08	3
Venlafaxine	N06AX16	Cetirizine	R06AE07	3
Citalopram	N06AB04	Pethidine	N02AB02	3
Venlafaxine	N06AX16	Levocetirizine	R06AE09	3
Nebivolol	C07AB12	Escitalopram	N06AB10	3
Phenprocoumon	B01AA04	Venlafaxine	N06AX16	3
Sumatriptan	N02CC01	Venlafaxine	N06AX16	3
Naratriptan	N02CC02	Venlafaxine	N06AX16	3
Edoxaban	B01AF03	Duloxetine	N06AX21	3
Empagliflozin	A10BK03	Duloxetine	N06AX21	3
Edoxaban	B01AF03	Citalopram	N06AB04	3
Dimenhydrinate	A04AB02	Citalopram	N06AB04	3
Dulaglutide	A10BJ05	Citalopram	N06AB04	3
Carvedilol	C07AG02	Citalopram	N06AB04	3
Diclofenac	D11AX18	Citalopram	N06AB04	3
Rivaroxaban	B01AF01	Sertraline	N06AB06	3
Diclofenac	D11AX18	Sertraline	N06AB06	3
Celecoxib	L01XX33	Sertraline	N06AB06	3
Tramadol	N02AJ13	Sertraline	N06AB06	3
Dalteparin natrium	B01AB04	Sertraline	N06AB06	3
Omeprazole	A02BC01	Fluoxetine	N06AB03	3
Ibuprofen	C01EB16	Paroxetine	N06AB05	3
Acetylsalicylic acid	A01AD05	Paroxetine	N06AB05	3
Glycopyrronium bromid	R03BB06	Opipramol	N06AA05	3
Buprenorphine	N02AE01	Amitriptyline	N06AA09	3
Tapentadol	N02AX06	Amitriptyline	N06AA09	3

Nebivolol	C07AB12	Bupropion	N06AX12	3
Phenprocoumon	B01AA04	Trazodone	N06AX05	3
Phenprocoumon	B01AA04	Valproic acid	N03AG01	3
Doxepine	D04AX01	Valproic acid	N03AG01	3
Buprenorphine	N02AE01	Carbamazepine	N03AF01	3
Paracetamol	N02BE01	Carbamazepine	N03AF01	3
Torasemide	C03CA04	Oxcarbazepine	N03AF02	3
Hydrochlorothiazid e	C03AA03	Oxcarbazepine	N03AF02	3
Topiramate	N02CX12	Oxcarbazepine	N03AF02	3
Dimenhydrinate	A04AB02	Quetiapine	N05AH04	3
Levomethadone	N02AC06	Quetiapine	N05AH04	3
Domperidone	A03FA03	Haloperidol	N05AD01	3
Doxepine	D04AX01	Haloperidol	N05AD01	3
Lithium	N05AN01	Torasemide	C03CA04	3
Bupropion	N06AX12	Prednisolone	A01AC04	2
Bupropion	N06AX12	Dexamethasone	A01AC02	2
TrImipramine	N06AA06	Hydrochlorothiazide	C03AA03	2
Fluoxetine	N06AB03	Hydrochlorothiazide	C03AA03	2
Clozapine	N05AH02	Omeprazole	A02BC01	2
Paroxetine	N06AB05	Omeprazole	A02BC01	2
Flupentixol	N05AF01	Pirenzepine	A02BX03	2
Olanzapine	N05AH03	Pirenzepine	A02BX03	2
Quetiapine	N05AH04	Pirenzepine	A02BX03	2
Paliperidone	N05AX13	Pirenzepine	A02BX03	2
Amitriptyline	N06AA09	Xipamide	C03BA10	2
Sertraline	N06AB06	Xipamide	C03BA10	2
Escitalopram	N06AB10	Xipamide	C03BA10	2
Venlafaxine	N06AX16	Xipamide	C03BA10	2
Sertraline	N06AB06	Metoclopramide	A03FA01	2
Amitriptyline	N06AA09	Indapamide	C03BA11	2
Trazodone	N06AX05	Indapamide	C03BA11	2
Sertraline	N06AB06	Dimenhydrinate	A04AB02	2
Bupropion	N06AX12	Dimenhydrinate	A04AB02	2
Clozapine	N05AH02	Medroxyprogesterone e	G03AA08	2
Clozapine	N05AH02	Chlortalidone	C03BA04	2
Fluvoxamine	N06AB08	Chlortalidone	C03BA04	2
Escitalopram	N06AB10	Chlortalidone	C03BA04	2
Carbamazepine	N03AF01	Verapamil	C08DA01	2
Risperidone	N05AX08	Verapamil	C08DA01	2
Fluoxetine	N06AB03	Tilidine	N02AX01	2
Paroxetine	N06AB05	Tilidine	N02AX01	2
Milnacipran	N06AX17	Tilidine	N02AX01	2
Escitalopram	N06AB10	Amiodarone	C01BD01	2
Amitriptyline	N06AA09	Celecoxib	L01XX33	2
Mirtazapine	N06AX11	Celecoxib	L01XX33	2
Citalopram	N06AB04	Ciprofloxacin	J01MA02	2
Duloxetine	N06AX21	Ciprofloxacin	J01MA02	2
Clozapine	N05AH02	Metamizole	M01BA07	2

Escitalopram	N06AB10	Oxycodone	N02AA05	2
Trazodone	N06AX05	Oxycodone	N02AA05	2
Sertraline	N06AB06	Buprenorphine	N02AE01	2
Bupropion	N06AX12	Chloroquine	P01BA01	2
Clozapine	N05AH02	Furosemide	C03CA01	2
Amitriptyline	N06AA09	Furosemide	C03CA01	2
Venlafaxine	N06AX16	Furosemide	C03CA01	2
Tranylcypromine	N06AF04	Bisoprolol	C07AB07	2
Moclobemide	N06AG02	Bisoprolol	C07AB07	2
Mirtazapine	N06AX11	Ethanol	B05XX04	2
Amitriptyline	N06AA09	Terbinafine	D01AE15	2
Mirtazapine	N06AX11	Clarithromycin	J01FA09	2
Clozapine	N05AH02	Candesartan	C09CA06	2
Venlafaxine	N06AX16	Chlorphenamine	R06AB04	2
Citalopram	N06AB04	Tapentadol	N02AX06	2
Sertraline	N06AB06	Tapentadol	N02AX06	2
Escitalopram	N06AB10	Tapentadol	N02AX06	2
Lamotrigine	N03AX09	Chlormadinone	G03AA15	2
Clozapine	N05AH02	Solifenacin	G04BD08	2
TrImipramine	N06AA06	Desloratadine	R06AX27	2
Mirtazapine	N06AX11	Desloratadine	R06AX27	2
Citalopram	N06AB04	Piritramide	N02AC03	2
Edoxaban	B01AF03	Escitalopram	N06AB10	2
Ticagrelor	B01AC24	Escitalopram	N06AB10	2
Liraglutide	A08AX02	Escitalopram	N06AB10	2
Ivabradine	C01EB17	Escitalopram	N06AB10	2
Naratriptan	N02CC02	Escitalopram	N06AB10	2
Dalteparin natrium	B01AB04	Escitalopram	N06AB10	2
Dabigatran	B01AE07	Escitalopram	N06AB10	2
Enoxaparin	B01AB05	Venlafaxine	N06AX16	2
Edoxaban	B01AF03	Venlafaxine	N06AX16	2
Amiodarone	C01BD01	Venlafaxine	N06AX16	2
Tamoxifen	L02BA01	Venlafaxine	N06AX16	2
Etoricoxib	M01AH05	Venlafaxine	N06AX16	2
Certoparin natrium	B01AB13	Venlafaxine	N06AX16	2
Ondansetron	A04AA01	Duloxetine	N06AX21	2
Saxagliptin	A10BD21	Duloxetine	N06AX21	2
Propafenone	C01BC03	Duloxetine	N06AX21	2
Celecoxib	L01XX33	Duloxetine	N06AX21	2
Sumatriptan	N02CC01	Duloxetine	N06AX21	2
Glimepiride	A10BB12	Citalopram	N06AB04	2
Domperidone	A03FA03	Citalopram	N06AB04	2
Granisetron	A04AA02	Citalopram	N06AB04	2
Saxagliptin	A10BD21	Citalopram	N06AB04	2
Tamoxifen	L02BA01	Citalopram	N06AB04	2
Tizanidine	M03BX02	Citalopram	N06AB04	2
Enoxaparin	B01AB05	Sertraline	N06AB06	2
Propranolol	C07AA05	Sertraline	N06AB06	2
Duloxetine	N06AX21	Sertraline	N06AB06	2

Doxepine	D04AX01	Fluoxetine	N06AB03	2
Sitagliptin	A10BD24	Paroxetine	N06AB05	2
Apixaban	B01AF02	Paroxetine	N06AB05	2
Clopidogrel	B01AC04	Paroxetine	N06AB05	2
Rivaroxaban	B01AF01	Paroxetine	N06AB05	2
Duloxetine	N06AX21	Paroxetine	N06AB05	2
Metformin	A10BA02	Fluvoxamine	N06AB08	2
Nebivolol	C07AB12	Fluvoxamine	N06AB08	2
Amantadine	J05AC04	Amitriptyline	N06AA09	2
Carvedilol	C07AG02	Bupropion	N06AX12	2
Phenprocoumon	B01AA04	Doxepine	N06AA12	2
Acetylsalicylic acid	A01AD05	Clomipramine	N06AA04	2
Lidocaine	A01AE01	Carbamazepine	N03AF01	2
Nitrendipine	C08CA08	Carbamazepine	N03AF01	2
Doxepine	D04AX01	Carbamazepine	N03AF01	2
Estradiol	D11AX34	Carbamazepine	N03AF01	2
Oxycodone	N02AA05	Carbamazepine	N03AF01	2
Duloxetine	N06AX21	Tranylcypromine	N06AF04	2
Doxepine	D04AX01	Aripiprazole	N05AX12	2
Clarithromycin	J01FA09	Aripiprazole	N05AX12	2
Indapamide	C03BA11	Quetiapine	N05AH04	2
Fluconazole	D01AC15	Quetiapine	N05AH04	2
Ciprofloxacin	J01MA02	Quetiapine	N05AH04	2
Glycopyrronium bromid	A03AB02	Quetiapine	N05AH04	2
Tramadol	N02AJ13	Pipamperone	N05AD05	2
Dimenhydrinate	A04AB02	Olanzapine	N05AH03	2
Trospium chloride	G04BD09	Olanzapine	N05AH03	2
Domperidone	A03FA03	Risperidone	N05AX08	2
Dimenhydrinate	A04AB02	Risperidone	N05AX08	2
Amantadine	J05AC04	Risperidone	N05AX08	2
Tramadol	N02AJ13	Risperidone	N05AX08	2
Trospium chloride	G04BD09	Risperidone	N05AX08	2
Indapamide	C03BA11	Clozapine	N05AH02	2
Indapamide	C03BA11	Prothipendyl	N05AX07	2
Indapamide	C03BA11	Amisulpride	N05AL05	2
Doxepine	D04AX01	Amisulpride	N05AL05	2
Dimenhydrinate	A04AB02	Melperon	N05AD03	2
Amiodarone	C01BD01	Melperon	N05AD03	2
Sotalol	C07AA07	Melperon	N05AD03	2
Trospium chloride	G04BD09	Melperon	N05AD03	2
Ondansetron	A04AA01	Haloperidol	N05AD01	2
Amiodarone	C01BD01	Haloperidol	N05AD01	2
Doxepine	D04AX01	Flupentixol	N05AF01	2
Clonidine	C02AC01	Mirtazapine	N06AX11	2
Amiodarone	C01BD01	Mirtazapine	N06AX11	2
Flecainide	C01BC04	Mirtazapine	N06AX11	2
Dronedarone	C01BD07	Mirtazapine	N06AX11	2
Brimonidine	D11AX21	Mirtazapine	N06AX11	2
Dasatinib	L01EA02	Mirtazapine	N06AX11	2

Tapentadol	N02AX06	TrImipramine	N06AA06	2
Doxepine	D04AX01	Paliperidone	N05AX13	2
Doxepine	D04AX01	Chlorprothixen	N05AF03	2
Brimonidine	D11AX21	Imipramine	N06AA02	2
Duloxetine	N06AX21	Moclobemide	N06AG02	2
Lithium	N05AN01	Verapamil	C08DA01	2
Lithium	N05AN01	Candesartan	C09CA06	2
Tryptophan	N06AX02	Duloxetine	N06AX21	1
Mianserin	N06AX03	Hydrochlorothiazide	C03AA03	1
Tianeptine	N06AX14	Hydrochlorothiazide	C03AA03	1
Clozapine	N05AH02	Esomeprazole	A02BC05	1
Citalopram	N06AB04	Esomeprazole	A02BC05	1
Fluphenazine	N05AB02	Pirenzepine	A02BX03	1
Benperidol	N05AD07	Pirenzepine	A02BX03	1
Chlorprothixen	N05AF03	Pirenzepine	A02BX03	1
Asenapine	N05AH05	Pirenzepine	A02BX03	1
Prothipendyl	N05AX07	Pirenzepine	A02BX03	1
Flupentixol	N05AF01	Atropine	A03BA01	1
Opipramol	N06AA05	Xipamide	C03BA10	1
Doxepine	N06AA12	Xipamide	C03BA10	1
Levomepromazine	N05AA02	Metoclopramide	A03FA01	1
Chlorprothixen	N05AF03	Metoclopramide	A03FA01	1
Zuclopenthixol	N05AF05	Metoclopramide	A03FA01	1
Olanzapine	N05AH03	Metoclopramide	A03FA01	1
Quetiapine	N05AH04	Metoclopramide	A03FA01	1
Sulpiride	N05AL01	Metoclopramide	A03FA01	1
Prothipendyl	N05AX07	Metoclopramide	A03FA01	1
Venlafaxine	N06AX16	Metoclopramide	A03FA01	1
Paroxetine	N06AB05	Indapamide	C03BA11	1
Sertraline	N06AB06	Indapamide	C03BA11	1
Escitalopram	N06AB10	Indapamide	C03BA11	1
Mirtazapine	N06AX11	Indapamide	C03BA11	1
Venlafaxine	N06AX16	Indapamide	C03BA11	1
Duloxetine	N06AX21	Indapamide	C03BA11	1
Venlafaxine	N06AX16	Dimenhydrinate	A04AB02	1
Melperon	N05AD03	Scopolamine	A04AD01	1
TrImipramine	N06AA06	Chlortalidone	C03BA04	1
Fluoxetine	N06AB03	Chlortalidone	C03BA04	1
Citalopram	N06AB04	Chlortalidone	C03BA04	1
Paroxetine	N06AB05	Chlortalidone	C03BA04	1
Lamotrigine	N03AX09	Drospirenone	G03AA12	1
Clozapine	N05AH02	Ethinylestradiol	G03CA01	1
Clozapine	N05AH02	Mesalazine	A07EC02	1
Clozapine	N05AH02	Allopurinol	M04AA01	1
Trazodone	N06AX05	Tilidine	N02AX01	1
Clozapine	N05AH02	Paracetamol	N02BE01	1
Escitalopram	N06AB10	Ciprofloxacin	J01MA02	1
Mirtazapine	N06AX11	Ciprofloxacin	J01MA02	1
Venlafaxine	N06AX16	Ciprofloxacin	J01MA02	1

Bupropion	N06AX12	Clopidogrel	B01AC04	1
Clozapine	N05AH02	Diclofenac	D11AX18	1
Clozapine	N05AH02	Naproxen	G02CC02	1
Mirtazapine	N06AX11	Enzalutamide	L02BB04	1
Lamotrigine	N03AX09	Estradiol	D11AX34	1
Clozapine	N05AH02	Estradiol	D11AX34	1
Moclobemide	N06AG02	Metoprolol	C07AB02	1
Fluoxetine	N06AB03	Oxycodone	N02AA05	1
Paroxetine	N06AB05	Oxycodone	N02AA05	1
Milnacipran	N06AX17	Oxycodone	N02AA05	1
Escitalopram	N06AB10	Fentanyl	N01AH01	1
Venlafaxine	N06AX16	Buprenorphine	N02AE01	1
Clomipramine	N06AA04	Chloroquine	P01BA01	1
Escitalopram	N06AB10	Chloroquine	P01BA01	1
Clomipramine	N06AA04	Dimetindene	D04AA13	1
Bupropion	N06AX12	Dimetindene	D04AA13	1
Bupropion	N06AX12	Ketotifen	R06AX17	1
Bupropion	N06AX12	Theophylline	C01EB28	1
Bupropion	N06AX12	Tramadol	N02AJ13	1
Imipramine	N06AA02	Torasemide	C03CA04	1
Clomipramine	N06AA04	Torasemide	C03CA04	1
Fluoxetine	N06AB03	Torasemide	C03CA04	1
Tianeptine	N06AX14	Torasemide	C03CA04	1
Reboxetine	N06AX18	Torasemide	C03CA04	1
Opipramol	N06AA05	Furosemide	C03CA01	1
Fluoxetine	N06AB03	Furosemide	C03CA01	1
Paroxetine	N06AB05	Furosemide	C03CA01	1
Sertraline	N06AB06	Furosemide	C03CA01	1
Trazodone	N06AX05	Furosemide	C03CA01	1
Milnacipran	N06AX17	Furosemide	C03CA01	1
TrImipramine	N06AA06	Piretanide	C03CA03	1
Mirtazapine	N06AX11	Piretanide	C03CA03	1
Clozapine	N05AH02	Trimethoprim	J01EA01	1
Escitalopram	N06AB10	Dronedarone	C01BD07	1
Moclobemide	N06AG02	Carvedilol	C07AG02	1
Valproic acid	N03AG01	Ethanol	B05XX04	1
Pipamperone	N05AD05	Ethanol	B05XX04	1
Doxepine	N06AA12	Ethanol	B05XX04	1
Escitalopram	N06AB10	Ethanol	B05XX04	1
Venlafaxine	N06AX16	Ethanol	B05XX04	1
Duloxetine	N06AX21	Ethanol	B05XX04	1
Haloperidol	N05AD01	Abiraterone	L02BX03	1
Amitriptyline	N06AA09	Abiraterone	L02BX03	1
Mirtazapine	N06AX11	Abiraterone	L02BX03	1
Fluoxetine	N06AB03	Flecainide	C01BC04	1
Sertraline	N06AB06	Flecainide	C01BC04	1
Clozapine	N05AH02	Spironolactone	C03DA01	1
Citalopram	N06AB04	Clarithromycin	J01FA09	1
Sertraline	N06AB06	Clarithromycin	J01FA09	1

Venlafaxine	N06AX16	Clarithromycin	J01FA09	1
Quetiapine	N05AH04	Piribedil	C04AX13	1
Mirtazapine	N06AX11	Diltiazem	C05AE03	1
Citalopram	N06AB04	Sotalol	C07AA07	1
Doxepine	N06AA12	Chlorphenamine	R06AB04	1
Quetiapine	N05AH04	Leuprorelin	H01CA04	1
Amitriptyline	N06AA09	Leuprorelin	H01CA04	1
Doxepine	N06AA12	Leuprorelin	H01CA04	1
Escitalopram	N06AB10	Leuprorelin	H01CA04	1
Mirtazapine	N06AX11	Leuprorelin	H01CA04	1
Doxepine	N06AA12	Loratadine	R06AX13	1
Paroxetine	N06AB05	Tapentadol	N02AX06	1
Milnacipran	N06AX17	Tapentadol	N02AX06	1
Duloxetine	N06AX21	Leflunomide	L04AA13	1
Quetiapine	N05AH04	Goserelin	H01CA05	1
Aripiprazole	N05AX12	Goserelin	H01CA05	1
Mirtazapine	N06AX11	Goserelin	H01CA05	1
Venlafaxine	N06AX16	Goserelin	H01CA05	1
Haloperidol	N05AD01	Tacrolimus	D11AH01	1
Fluphenazine	N05AB02	Bromocriptine	G02CB01	1
Quetiapine	N05AH04	Bromocriptine	G02CB01	1
Paliperidone	N05AX13	Bromocriptine	G02CB01	1
Lamotrigine	N03AX09	Levonorgestrel	G03AA07	1
Lamotrigine	N03AX09	Desogestrel	G03AA09	1
Clozapine	N05AH02	Desogestrel	G03AA09	1
Lamotrigine	N03AX09	Ritonavir	J05AE03	1
Venlafaxine	N06AX16	Ritonavir	J05AE03	1
Melperon	N05AD03	Propiverine	G04BD06	1
Pipamperone	N05AD05	Propiverine	G04BD06	1
Clozapine	N05AH02	Propiverine	G04BD06	1
Quetiapine	N05AH04	Propiverine	G04BD06	1
Risperidone	N05AX08	Propiverine	G04BD06	1
Paliperidone	N05AX13	Propiverine	G04BD06	1
Quetiapine	N05AH04	Tolterodine	G04BD07	1
Prothipendyl	N05AX07	Tolterodine	G04BD07	1
Quetiapine	N05AH04	Solifenacin	G04BD08	1
Perazine	N05AB10	Darifenacin	G04BD10	1
Venlafaxine	N06AX16	Darifenacin	G04BD10	1
Clozapine	N05AH02	Apomorphine	G04BE07	1
Quetiapine	N05AH04	Apomorphine	G04BE07	1
Haloperidol	N05AD01	Methocarbamol	M03BA03	1
Pipamperone	N05AD05	Methocarbamol	M03BA03	1
Zuclopenthixol	N05AF05	Methocarbamol	M03BA03	1
Melperon	N05AD03	Hydroxychloroquine	P01BA02	1
Olanzapine	N05AH03	Hydroxychloroquine	P01BA02	1
Quetiapine	N05AH04	Hydroxychloroquine	P01BA02	1
Risperidone	N05AX08	Hydroxychloroquine	P01BA02	1
Citalopram	N06AB04	Hydroxychloroquine	P01BA02	1
Clozapine	N05AH02	Piperacillin	J01CA12	1

Clozapine	N05AH02	Fosfomycin	J01XX01	1
Citalopram	N06AB04	Moxifloxacin	J01MA14	1
Amitriptyline	N06AA09	Cetirizine	R06AE07	1
Trazodone	N06AX05	Cetirizine	R06AE07	1
Amitriptyline	N06AA09	Doxylamin	R06AA09	1
Fluoxetine	N06AB03	Alfentanil	N01AH02	1
Trazodone	N06AX05	Rupatadine	R06AX28	1
Mirtazapine	N06AX11	Fexofenadine	R06AX26	1
Mirtazapine	N06AX11	Mefloquine	P01BC02	1
Venlafaxine	N06AX16	Hydroxyzine	R06AX33	1
Glimepiride	A10BB12	Escitalopram	N06AB10	1
Dimenhydrinate	A04AB02	Escitalopram	N06AB10	1
Vildagliptin	A10BH02	Escitalopram	N06AB10	1
Dipyridamol	B01AC07	Escitalopram	N06AB10	1
Propranolol	C07AA05	Escitalopram	N06AB10	1
Doxepine	D04AX01	Escitalopram	N06AB10	1
Amantadine	J05AC04	Escitalopram	N06AB10	1
Fingolimod	L04AA27	Escitalopram	N06AB10	1
Etoricoxib	M01AH05	Escitalopram	N06AB10	1
Sumatriptan	N02CC01	Escitalopram	N06AB10	1
Nadroparin calcium	B01AB06	Escitalopram	N06AB10	1
Glimepiride	A10BB12	Venlafaxine	N06AX16	1
Flecainide	C01BC04	Venlafaxine	N06AX16	1
Dulaglutide	A10BJ05	Venlafaxine	N06AX16	1
Exenatide	A10BJ01	Venlafaxine	N06AX16	1
Dapagliflozin	A10BK01	Venlafaxine	N06AX16	1
Ivabradine	C01EB17	Venlafaxine	N06AX16	1
Prasugrel	B01AC22	Venlafaxine	N06AX16	1
Amantadine	J05AC04	Venlafaxine	N06AX16	1
Celecoxib	L01XX33	Venlafaxine	N06AX16	1
Fingolimod	L04AA27	Venlafaxine	N06AX16	1
Rizatriptan	N02CC04	Venlafaxine	N06AX16	1
Dextromethorphan	R05DA09	Venlafaxine	N06AX16	1
Dalteparin natrium	B01AB04	Venlafaxine	N06AX16	1
Enoxaparin	B01AB05	Duloxetine	N06AX21	1
Flecainide	C01BC04	Duloxetine	N06AX21	1
Semaglutide	A10BJ06	Duloxetine	N06AX21	1
Prasugrel	B01AC22	Duloxetine	N06AX21	1
Timolol	C07AA06	Duloxetine	N06AX21	1
Tamoxifen	L02BA01	Duloxetine	N06AX21	1
Acemetacin	M01AB11	Duloxetine	N06AX21	1
Dexibuprofen	M01AE14	Duloxetine	N06AX21	1
Almotriptan	N02CC05	Duloxetine	N06AX21	1
Heparin	B01AB01	Duloxetine	N06AX21	1
Certoparin natrium	B01AB13	Duloxetine	N06AX21	1
Fondaparinux natrium	B01AX05	Duloxetine	N06AX21	1
Ondansetron	A04AA01	Citalopram	N06AB04	1
Dapagliflozin	A10BK01	Citalopram	N06AB04	1

Repaglinide	A10BX02	Citalopram	N06AB04	1
Indometacin	C01EB03	Citalopram	N06AB04	1
Quinine	C05AF01	Citalopram	N06AB04	1
Propranolol	C07AA05	Citalopram	N06AB04	1
Timolol	C07AA06	Citalopram	N06AB04	1
Naproxen	G02CC02	Citalopram	N06AB04	1
Amantadine	J05AC04	Citalopram	N06AB04	1
Celecoxib	L01XX33	Citalopram	N06AB04	1
Etoricoxib	M01AH05	Citalopram	N06AB04	1
Sumatriptan	N02CC01	Citalopram	N06AB04	1
Heparin	B01AB01	Citalopram	N06AB04	1
Glimepiride	A10BB12	Sertraline	N06AB06	1
Ondansetron	A04AA01	Sertraline	N06AB06	1
Empagliflozin	A10BK03	Sertraline	N06AB06	1
Dulaglutide	A10BJ05	Sertraline	N06AB06	1
Saxagliptin	A10BD21	Sertraline	N06AB06	1
Tinzaparin	B01AB10	Sertraline	N06AB06	1
Carvedilol	C07AG02	Sertraline	N06AB06	1
Nilotinib	L01EA03	Sertraline	N06AB06	1
Tamoxifen	L02BA01	Sertraline	N06AB06	1
Sumatriptan	N02CC01	Sertraline	N06AB06	1
Rizatriptan	N02CC04	Sertraline	N06AB06	1
Heparin	B01AB01	Sertraline	N06AB06	1
Apixaban	B01AF02	Milnacipran	N06AX17	1
Naproxen	G02CC02	Milnacipran	N06AX17	1
Esomeprazole	A02BC05	Fluoxetine	N06AB03	1
Nebivolol	C07AB12	Fluoxetine	N06AB03	1
Carvedilol	C07AG02	Fluoxetine	N06AB03	1
Diclofenac	D11AX18	Fluoxetine	N06AB03	1
Etoricoxib	M01AH05	Fluoxetine	N06AB03	1
Codeine	N02AJ05	Fluoxetine	N06AB03	1
Tramadol	N02AJ13	Fluoxetine	N06AB03	1
Phenprocoumon	B01AA04	Paroxetine	N06AB05	1
Empagliflozin	A10BK03	Paroxetine	N06AB05	1
Flecainide	C01BC04	Paroxetine	N06AB05	1
Propranolol	C07AA05	Paroxetine	N06AB05	1
Doxepine	D04AX01	Paroxetine	N06AB05	1
Desmopressin	H01BA02	Paroxetine	N06AB05	1
Tramadol	N02AJ13	Paroxetine	N06AB05	1
Ibuprofen	C01EB16	Fluvoxamine	N06AB08	1
Sitagliptin	A10BD24	Fluvoxamine	N06AB08	1
Omeprazole	A02BC01	Fluvoxamine	N06AB08	1
Glimepiride	A10BB12	Fluvoxamine	N06AB08	1
Saxagliptin	A10BD21	Fluvoxamine	N06AB08	1
Naproxen	G02CC02	Fluvoxamine	N06AB08	1
Duloxetine	N06AX21	Fluvoxamine	N06AB08	1
Clonidine	C02AC01	Opipramol	N06AA05	1
Tramadol	N02AJ13	Opipramol	N06AA05	1
Trospium chloride	G04BD09	Opipramol	N06AA05	1

Domperidone	A03FA03	Amitriptyline	N06AA09	1
Moxonidine	C02AC05	Amitriptyline	N06AA09	1
Warfarin	B01AA03	Amitriptyline	N06AA09	1
Dopamine	C01CA04	Amitriptyline	N06AA09	1
Propiverine	G04BD06	Amitriptyline	N06AA09	1
Trospium chloride	G04BD09	Amitriptyline	N06AA09	1
Timolol	C07AA06	Bupropion	N06AX12	1
Amantadine	J05AC04	Bupropion	N06AX12	1
Moxonidine	C02AC05	Trazodone	N06AX05	1
Ranolazine	C01EB18	Trazodone	N06AX05	1
Levomethadone	N02AC06	Trazodone	N06AX05	1
Tramadol	N02AJ13	Trazodone	N06AX05	1
Clonidine	C02AC01	Doxepine	N06AA12	1
Pridinol	M03BX03	Doxepine	N06AA12	1
Fentanyl	N01AH01	Doxepine	N06AA12	1
Buprenorphine	N02AE01	Doxepine	N06AA12	1
Glycopyrronium bromid	A03AB02	Doxepine	N06AA12	1
Trospium chloride	G04BD09	Doxepine	N06AA12	1
Phenprocoumon	B01AA04	Clomipramine	N06AA04	1
Flecainide	C01BC04	Clomipramine	N06AA04	1
Naproxen	G02CC02	Clomipramine	N06AA04	1
Desmopressin	H01BA02	Clomipramine	N06AA04	1
Trospium chloride	G04BD09	Clomipramine	N06AA04	1
Doxycycline	A01AB22	Carbamazepine	N03AF01	1
Hydrocortisone	A01AC03	Carbamazepine	N03AF01	1
Betamethasone	A01AC05	Carbamazepine	N03AF01	1
Clopidogrel	B01AC04	Carbamazepine	N03AF01	1
Rivaroxaban	B01AF01	Carbamazepine	N03AF01	1
Xipamide	C03BA10	Carbamazepine	N03AF01	1
Edoxaban	B01AF03	Carbamazepine	N03AF01	1
Prednisone	A07EA03	Carbamazepine	N03AF01	1
Budesonide	A07EA06	Carbamazepine	N03AF01	1
Eplerenone	C03DA04	Carbamazepine	N03AF01	1
Nifedipine	C08CA05	Carbamazepine	N03AF01	1
Ketoconazole	D01AC08	Carbamazepine	N03AF01	1
Norethisterone	G03AA05	Carbamazepine	N03AF01	1
Desogestrel	G03AA09	Carbamazepine	N03AF01	1
Etonogestrel	G03AC08	Carbamazepine	N03AF01	1
Progesterone	G03DA04	Carbamazepine	N03AF01	1
Imatinib	L01EA01	Carbamazepine	N03AF01	1
Fingolimod	L04AA27	Carbamazepine	N03AF01	1
Fentanyl	N01AH01	Carbamazepine	N03AF01	1
Morphine	N02AA01	Carbamazepine	N03AF01	1
Levomethadone	N02AC06	Carbamazepine	N03AF01	1
Montelukast	R03DC03	Carbamazepine	N03AF01	1
Roflumilast	R03DX07	Carbamazepine	N03AF01	1
Liothyronine	H03AA02	Carbamazepine	N03AF01	1
Sitagliptin	A10BD24	Tranylcypromine	N06AF04	1
Empagliflozin	A10BK03	Tranylcypromine	N06AF04	1

Buprenorphine	N02AE01	Tranylcypromine	N06AF04	1
Domperidone	A03FA03	Quetiapine	N05AH04	1
Amiodarone	C01BD01	Quetiapine	N05AH04	1
Flecainide	C01BC04	Quetiapine	N05AH04	1
Roxithromycin	J01FA06	Quetiapine	N05AH04	1
Clarithromycin	J01FA09	Quetiapine	N05AH04	1
Tizanidine	M03BX02	Quetiapine	N05AH04	1
Ondansetron	A04AA01	Pipamperone	N05AD05	1
Dimenhydrinate	A04AB02	Pipamperone	N05AD05	1
Flecainide	C01BC04	Pipamperone	N05AD05	1
Indapamide	C03BA11	Pipamperone	N05AD05	1
Ivabradine	C01EB17	Pipamperone	N05AD05	1
Levomethadone	N02AC06	Pipamperone	N05AD05	1
Glycopyrronium bromid	A03AB02	Pipamperone	N05AD05	1
Roxithromycin	J01FA06	Olanzapine	N05AH03	1
Fingolimod	L04AA27	Olanzapine	N05AH03	1
Tramadol	N02AJ13	Olanzapine	N05AH03	1
Glycopyrronium bromid	A03AB02	Olanzapine	N05AH03	1
Ondansetron	A04AA01	Risperidone	N05AX08	1
Granisetron	A04AA02	Risperidone	N05AX08	1
Ciprofloxacin	J01MA02	Risperidone	N05AX08	1
Glycopyrronium bromid	A03AB02	Risperidone	N05AX08	1
Ranolazine	C01EB18	Clozapine	N05AH02	1
Doxepine	D04AX01	Clozapine	N05AH02	1
Levomethadone	N02AC06	Clozapine	N05AH02	1
Trospium chloride	G04BD09	Clozapine	N05AH02	1
Doxepine	D04AX01	Prothipendyl	N05AX07	1
Ciprofloxacin	J01MA02	Prothipendyl	N05AX07	1
Levomethadone	N02AC06	Prothipendyl	N05AX07	1
Trospium chloride	G04BD09	Prothipendyl	N05AX07	1
Amantadine	J05AC04	Amisulpride	N05AL05	1
Glycopyrronium bromid	A03AB02	Amisulpride	N05AL05	1
Domperidone	A03FA03	Melperon	N05AD03	1
Granisetron	A04AA02	Melperon	N05AD03	1
Flecainide	C01BC04	Melperon	N05AD03	1
Indapamide	C03BA11	Melperon	N05AD03	1
Clarithromycin	J01FA09	Melperon	N05AD03	1
Ciprofloxacin	J01MA02	Melperon	N05AD03	1
Tizanidine	M03BX02	Melperon	N05AD03	1
Hydroxyzine	R06AX33	Melperon	N05AD03	1
Dimenhydrinate	A04AB02	Haloperidol	N05AD01	1
Roxithromycin	J01FA06	Haloperidol	N05AD01	1
Amantadine	J05AC04	Flupentixol	N05AF01	1
Levomethadone	N02AC06	Flupentixol	N05AF01	1
Dimenhydrinate	A04AB02	Sulpiride	N05AL01	1
Doxepine	D04AX01	Sulpiride	N05AL01	1

Ranolazine	C01EB18	Mirtazapine	N06AX11	1
Quinine	C05AF01	Mirtazapine	N06AX11	1
Desmopressin	H01BA02	Mirtazapine	N06AX11	1
Levomethadone	N02AC06	Mirtazapine	N06AX11	1
Clonidine	C02AC01	TrImipramine	N06AA06	1
Oxybutynin	G04BD04	TrImipramine	N06AA06	1
Tizanidine	M03BX02	TrImipramine	N06AA06	1
Levomethadone	N02AC06	TrImipramine	N06AA06	1
Buprenorphine	N02AE01	TrImipramine	N06AA06	1
Tramadol	N02AJ13	TrImipramine	N06AA06	1
Trospium chloride	G04BD09	TrImipramine	N06AA06	1
Ondansetron	A04AA01	Levomepromazine	N05AA02	1
Dimenhydrinate	A04AB02	Levomepromazine	N05AA02	1
Indapamide	C03BA11	Levomepromazine	N05AA02	1
Doxepine	D04AX01	Levomepromazine	N05AA02	1
Amantadine	J05AC04	Levomepromazine	N05AA02	1
Trospium chloride	G04BD09	Levomepromazine	N05AA02	1
Dimenhydrinate	A04AB02	Fluphenazine	N05AB02	1
Dimenhydrinate	A04AB02	Paliperidone	N05AX13	1
Amiodarone	C01BD01	Paliperidone	N05AX13	1
Ivabradine	C01EB17	Paliperidone	N05AX13	1
Dimenhydrinate	A04AB02	Zuclopenthixol	N05AF05	1
Rilpivirine	J05AG05	Zuclopenthixol	N05AF05	1
Fingolimod	L04AA27	Zuclopenthixol	N05AF05	1
Fingolimod	L04AA27	Chlorprothixen	N05AF03	1
Propiverine	G04BD06	Imipramine	N06AA02	1
Tramadol	N02AJ13	Imipramine	N06AA02	1
Tilidine	N02AX01	Imipramine	N06AA02	1
Fingolimod	L04AA27	Maprotiline	N06AA21	1
Tramadol	N02AJ13	Maprotiline	N06AA21	1
Dimenhydrinate	R06AA11	Lithium	N05AN01	1
Ivabradine	C01EB17	Lithium	N05AN01	1
Clarithromycin	J01FA09	Lithium	N05AN01	1
Lithium	N05AN01	Hydrochlorothiazide	C03AA03	1
Lithium	N05AN01	Ibuprofen	M01AE01	1
Lithium	N05AN01	Furosemide	C03CA01	1
Lithium	N05AN01	Spironolactone	C03DA01	1
Lithium	N05AN01	Valsartan	C09CA03	1
Lithium	N05AN01	Losartan	C09CA01	1
