# Supplementary material for: Analysis of drug-drug interactions in spontaneous adverse drug reaction reports from EudraVigilance focusing on psychiatric drugs and somatic medication
Source: BMC Psychiatry. 2025 Oct 2;25:914. doi: 10.1186/s12888-025-07352-8 (PMC12490046; doi:10.1186/s12888-025-07352-8)
Supplement: Supplementary file 3 — Supplementary Material 3. [file 12888_2025_7352_MOESM3_ESM.pdf]

Additional file 3) Sex analysis: mean number of pDDI and mean number of drugs per ADR report depending on patient age.

3.1 Analysis of reports referring to females

Figure 1) Mean number of pDDI per ADR report depending on patient age.

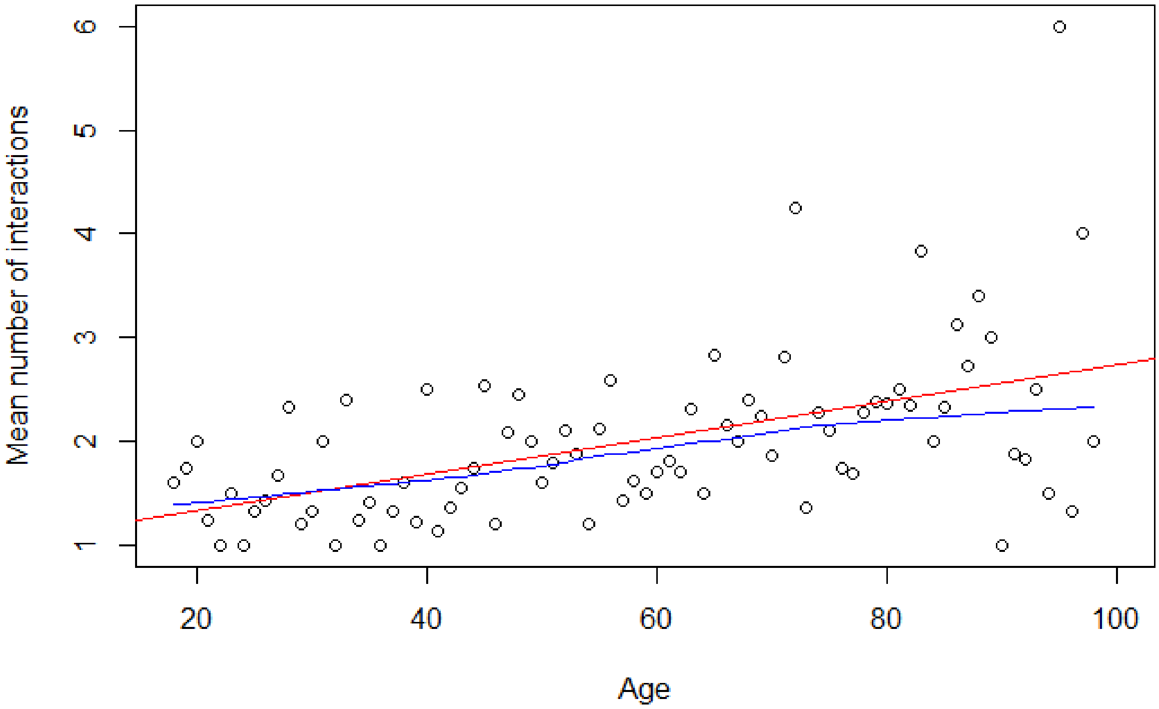

An increase of the mean number of pDDI per ADR report depending on patient age was observed. The red line shows the regression line and the blue line the fitted regression line.

Figure 2) Mean number of drugs reported as suspected/interacting or concomitant per ADR depending on patient age.

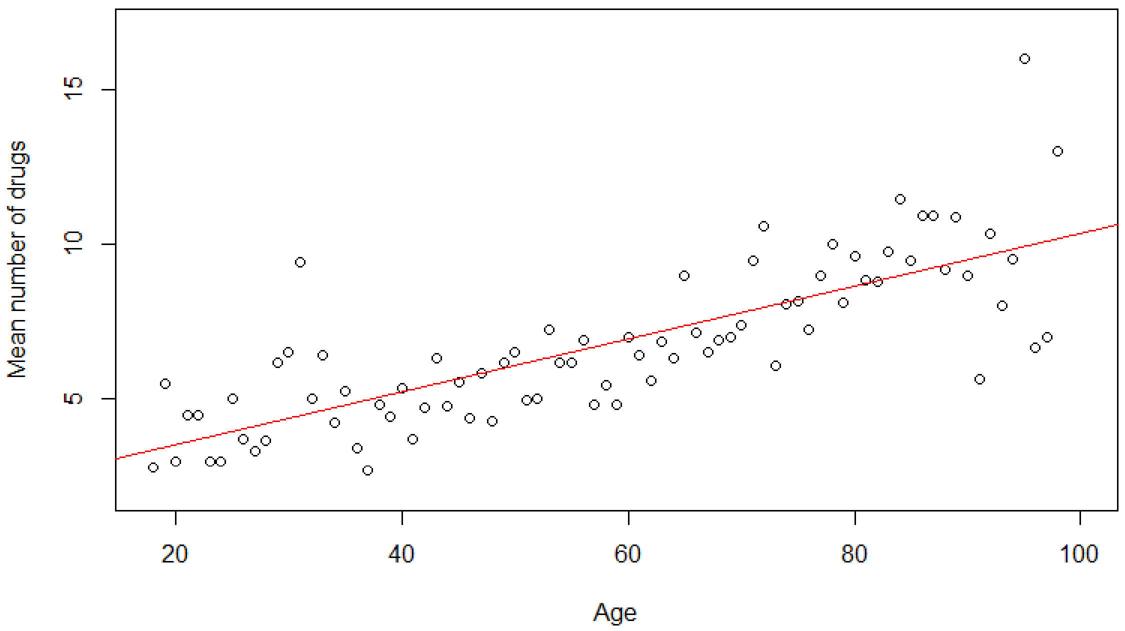

An increase of the mean number of drugs reported as suspected/interacting or concomitant per ADR report was observed.

3.1 Analysis of reports referring to males

Figure 3) Mean number of pDDI per ADR report depending on patient age.

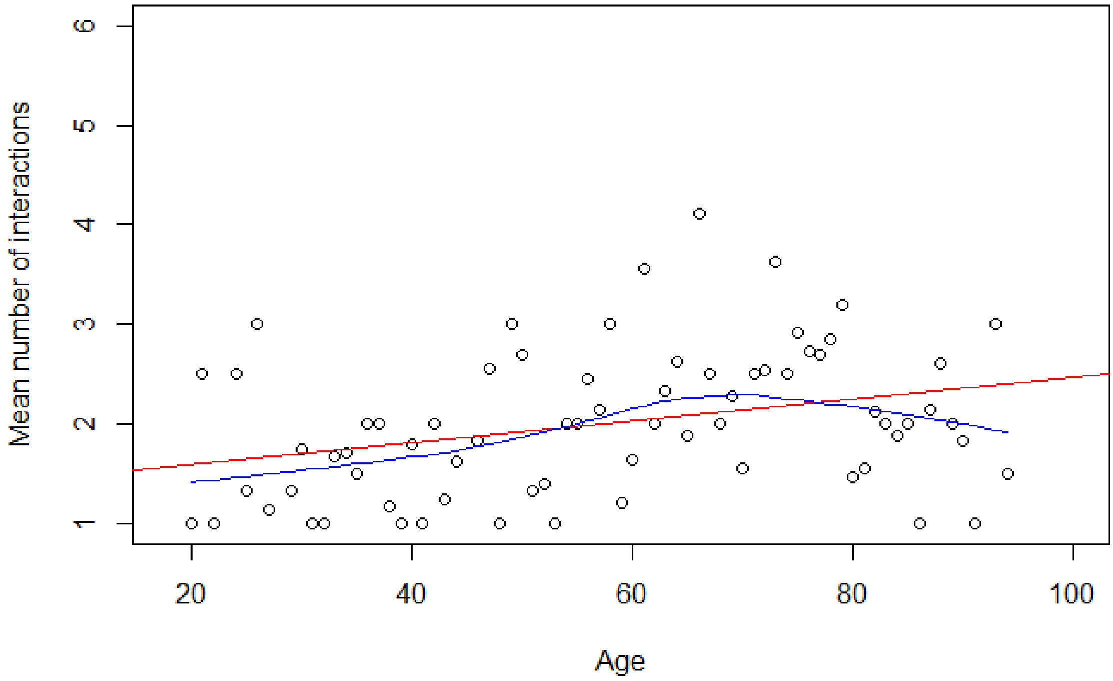

An increase of the mean number of pDDI per ADR report depending on patient age was observed until the age of 70 years. Afterwards the fitted regression line shows a slight decrease of the mean number of pDDI per ADR report. The red line shows the regression line and the blue line the fitted regression line.

Figure 4) Mean number of drugs reported as suspected/interacting or concomitant per ADR report depending on patient age.

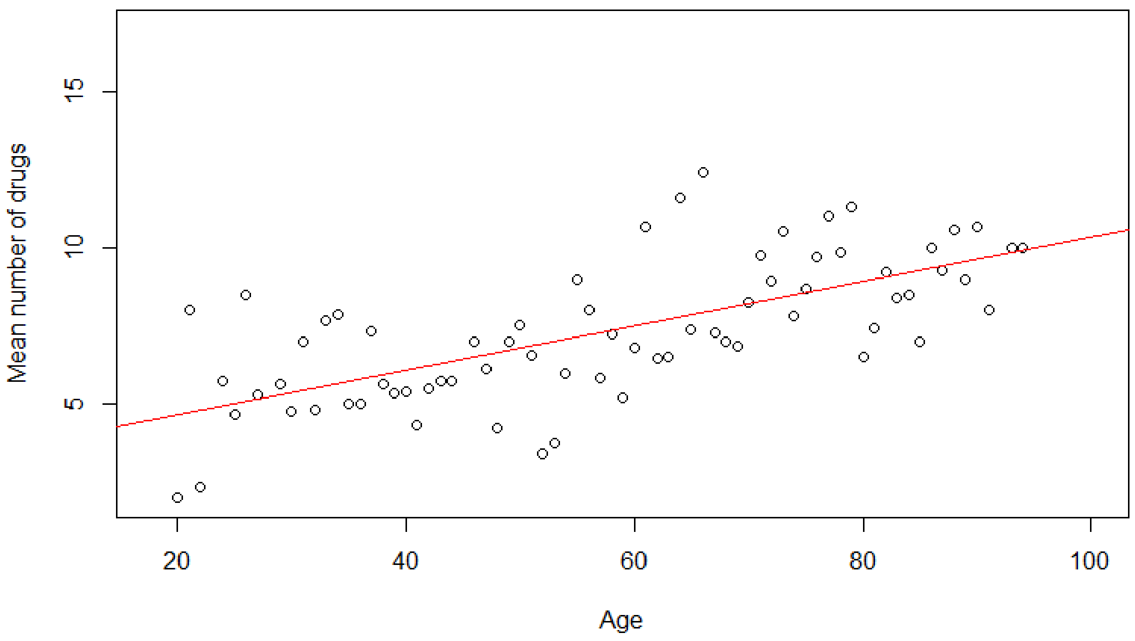

An increase of the mean number of drugs reported as suspected/interacting or concomitant per ADR report was observed.
