# Supplementary material for: Analysis of drug-drug interactions in spontaneous adverse drug reaction reports from EudraVigilance focusing on psychiatric drugs and somatic medication
Source: BMC Psychiatry. 2025 Oct 2;25:914. doi: 10.1186/s12888-025-07352-8 (PMC12490046; doi:10.1186/s12888-025-07352-8)
Supplement: Supplementary file 4 — Supplementary Material 4. [file 12888_2025_7352_MOESM4_ESM.pdf]

Additional file 4) Grouping by pharmacological effects and drug classes of the potentially interacting drug pairs with more than 10 reports.

Pharmacological effects/ADRs according to ABDATA	Potentially interacting drug pairs according to ABDATA	Number of reports[1]	Grouping of drug classes[2]
Bleeding events	Acetylsalicylic acid - Citalopram	55	SSRIs – Platelet aggregation inhibitors
Bleeding events	Acetylsalicylic acid - Duloxetine	41	SSRIs – Platelet aggregation inhibitors
Bleeding events	Acetylsalicylic acid - Venlafaxine	40	SSRIs – Platelet aggregation inhibitors
Bleeding events	Acetylsalicylic acid - Escitalopram	30	SSRIs – Platelet aggregation inhibitors
Bleeding events	Acetylsalicylic acid - Sertraline	28	SSRIs – Platelet aggregation inhibitors
Bleeding events	Citalopram - Clopidogrel	12	SSRIs – Platelet aggregation inhibitors
Bleeding events	Duloxetine - Ibuprofen	16	SSRIs - NSAIDs
Bleeding events	Citalopram - Ibuprofen	15	SSRIs - NSAIDs
Bleeding events	Ibuprofen - Venlafaxine	15	SSRIs - NSAIDs
Bleeding events	Apixaban - Citalopram	20	SSRIs - Anticoagulants
Bleeding events	Apixaban - Duloxetine	12	SSRIs - Anticoagulants
Bleeding events	Apixaban - Escitalopram	11	SSRIs - Anticoagulants

Granulocytopenia/agranulocytosis	Clozapine - Pantoprazole	26	Clozapine – ASS/ramipril/Pantoprazole
Granulocytopenia/agranulocytosis	Acetylsalicylic acid - Clozapine	12	Clozapine – ASS/ramipril/Pantoprazole
Granulocytopenia/agranulocytosis	Clozapine - Ramipril	12	Clozapine – ASS/ramipril/Pantoprazole
Hyperammonic encephalopathy	Topiramate - Valproinic acid	15	Valproinic acid - Topiramate
Hypo- or hyperglycemia	Metformin - Venlafaxine	24	SSRIs - Antidiabetics
Hypo- or hyperglycemia	Duloxetine - Metformin	23	SSRIs - Antidiabetics
Hypo- or hyperglycemia	Citalopram - Metformin	15	SSRIs - Antidiabetics
Hypo- or hyperglycemia	Metformin - Sertraline	12	SSRIs - Antidiabetics
Hypo- or hyperglycemia	Duloxetine - Sitagliptin	11	SSRIs - Antidiabetics
Hyponatremia	Mirtazapine - Torasemide	71	Antidepressants - Antidiuretics
Hyponatremia	Citalopram - Torasemide	37	Antidepressants - Antidiuretics
Hyponatremia	Hydrochlorothiazide - Mirtazapine	33	Antidepressants - Antidiuretics
Hyponatremia	Duloxetine - Torasemide	28	Antidepressants - Antidiuretics
Hyponatremia	Torasemide - Venlafaxine	25	Antidepressants - Antidiuretics
Hyponatremia	Sertraline - Torasemide	24	Antidepressants - Antidiuretics
Hyponatremia	Citalopram - Hydrochlorothiazide	22	Antidepressants - Antidiuretics

Hyponatremia	Hydrochlorothiazide - Venlafaxine	22	Antidepressants - Antidiuretics
Hyponatremia	Duloxetine - Hydrochlorothiazide	19	Antidepressants - Antidiuretics
Hyponatremia	Escitalopram - Torasemide	17	Antidepressants - Antidiuretics
Hyponatremia	Escitalopram - Hydrochlorothiazide	16	Antidepressants - Antidiuretics
Hyponatremia	Hydrochlorothiazide - Sertraline	15	Antidepressants - Antidiuretics
Hyponatremia	Amitryptiline - Torasemide	11	Antidepressants - Antidiuretics
Hyponatremia	Citalopram - Furosemide	11	Antidepressants - Antidiuretics
Hyponatremia	Furosemide - Mirtazapine	11	Antidepressants - Antidiuretics
Hypothyroidism	Carbamazepine - Levothyroxine	16	Levothyroxine - Carbamazepine
Increased anticholinergic effects	Clozapine - Pirenzepine	26	Clozapine - Pirenzepine
Increased beta-blocker effects	Citalopram - Metoprolol	45	SSRIs – Beta-blockers
Increased beta-blocker effects	Duloxetine - Metoprolol	33	SSRIs – Beta-blockers
Increased beta-blocker effects	Escitalopram - Metoprolol	27	SSRIs – Beta-blockers
Increased beta-blocker effects	Metoprolol - Sertraline	21	SSRIs – Beta-blockers
Increased mortality with dementia	Furosemide - Risperidone	12	Furosemide - Risperidone
Reduced efficacy of CYP substrats	Metamizole - Sertraline	18	Metamizole – Sertraline/valproinic acid
Reduced efficacy of CYP substrats	Metamizole - Valproinic acid	17	Metamizole – Sertraline/valproinic acid

Serotonin syndrome	Duloxetine - Tilidine	17	Serotonergic antidepressants - Serotonergic opioids
Serotonin syndrome	Amitryptiline - Oxycodone	13	Serotonergic antidepressants - Serotonergic opioids
Serotonin syndrome	Tilidine - Venlafaxine	13	Serotonergic antidepressants - Serotonergic opioids
Serotonin syndrome	Citalopram - Oxycodone	12	Serotonergic antidepressants - Serotonergic opioids
Serotonin syndrome	Duloxetine - Oxycodone	12	Serotonergic antidepressants - Serotonergic opioids
Toxicity to valproinic acid and increased bleeding time	Acetylsalicylic acid - Valproinic acid	22	Valproinic acid - ASS

[1] note that more than one potential drug-drug interaction (pDDI) could be identified per ADR report. Thus, the sum of the number of reports reflect the number of pDDI and may exceed the number of reports.

[2] note that the grouping of drug classes was performed based on the reports with potentially interacting drug pairs with more than 10 reports. Thus, the drug classes in our analysis are not a complete presentation of these drug classes.
