# Supplementary material for: Analysis of drug-drug interactions in spontaneous adverse drug reaction reports from EudraVigilance focusing on psychiatric drugs and somatic medication
Source: BMC Psychiatry. 2025 Oct 2;25:914. doi: 10.1186/s12888-025-07352-8 (PMC12490046; doi:10.1186/s12888-025-07352-8)
Supplement: Supplementary file 5 — Supplementary Material 5. [file 12888_2025_7352_MOESM5_ESM.pdf]

Additional file 5) Calculation of additive risk in the database.

Methods: We identified all spontaneous reports from Germany received between 01/2017-12/2021 for patients older than 17 years and excluded all spontaneous reports related to vaccines and medicines for the use of hyposensitization (n= 273,309 reports remained).

The following procedure is now described using the example hyponatremia (specific pharmacological effect) related to antidepressants (first drug class) and antidiuretics (second drug class). At first, the number of reports describing the specific pharmacological effect (e.g. hyponatremia, n= 430), and the number of reports explicitly related to the respective drug classes (e.g. antidepressants (n= 3,214) and antidiuretics (n= 724)) were identified. After that, the number of reports with the specific pharmacological effect (e.g. hyponatremia) explicitly related to the first drug class (e.g. antidepressants, n= 6) and explicitly related the second drug class (e.g. antidiuretics, n= 5) of the potentially interacting drug pair was determined. The number of reports describing the specific pharmacological effect (e.g. hyponatremia) related to the potentially interacting drug pair (e.g. antidepressants and antidiuretics) was already determined through the individual case assessment (see manuscript figure 5) (n= 22). Thus, the number of reports related to all other drugs exclusive the analysed drug classes (e.g. antidepressants and antidiuretics) results from the difference between the previously determined numbers (e.g. 430 – 22 – 6 – 5 = 397). However, it is important to note that our drug classes are not a complete representation of these drug classes since our analysis was restricted to potentially interacting drug pairs with more than 10 reports in order to focus on the most important potentially interacting drug pairs in our analysis. In case of the example of antidepressants and antidiuretics, this means that our group of antidepressants consists of citalopram, escitalopram, duloxetine, mirtazapine, sertraline, venlafaxine, amitriptyline and our group of antidiuretics consist of hydrochlorothiazide, furosemide, torasemide and their combination products. The composition of the respective drug classes can be found in the legend below the table.

Table 1) Raw data of the number of reports in the database.

ADR - potentially interacting drug pairs[1]	Number of reports with DDI/number of reports related to the potential interacting drug pair	Number of reports with the respective ADR to the first drug of the respective potentially interacting drug pair/number of reports related to the first drug	Number of reports with the respective ADR to the second drug of the respective potentially interacting drug pair/number of reports related to the second drug of the	Number of reports with the respective ADR to all other drugs exclusive the respective potentially interacting drug pair/number of reports related to all other drugs exclusive the

		of the respective potentially interacting drug pair	respective potentially interacting drug pair	respective potentially interacting drug pair
Hyponatremia – antidepressants and antidiuretics[2]	7.6% (22/289)	0.2% (6/3214)	0.7% (5/724)	0.1% (397/272912)
Bleeding events – SSRIs and platelet aggregation inhibitors[3]	23.7% (45/190)	1.6% (40/2558)	16.7% (172/1030)	6.6% (17938/273325)
Bleeding events – SSRIs and anticoagulants[4]	33.3% (14/42)	1.7% (24/1380)	33.4% (1088/3253)	6.3% (17069/272428)
Bleeding events – SSRIs and NSAIDs[5]	17.4% (8/46)	1.1% (17/1572)	4.2% (54/1275)	6.6% (18116/274210)
Increased effect of beta-blockers – SSRIs and beta-blockers[6]	6.5% (8/124)	0.4% (8/1824)	3.1% (27/872)	1.3% (3688/274283)
Hypo- and hyperglycemia – SSRIs and antidiabetics[7]	9.1% (7/77)	0.2% (4/2016)	4.8% (61/1264)	2.1% (5720/273746)
Serotonin syndrome – serotonergic antidepressants and opioids[8]	5.3% (3/57)	0.4% (8/1826)	0.0% (0/171)	0.0% (72/275049)

[1] the analysis is based on the reports of the drugs of the potentially interacting drug pairs with more than 10 reports. Thus, the drug classes in our analysis are not a complete presentation of these drug classes.

[2] the drug class of antidepressants consists of citalopram, escitalopram, duloxetine, mirtazapine, sertraline, venlafaxine, amitriptyline and the drug class of antidiuretics consist of hydrochlorothiazide, furosemide, torasemide and their combination products.

[3] the drug class of SSRIs consists of citalopram, sertraline, escitalopram, venlafaxine, duloxetine and the drug class of platelet aggregation inhibitors consist of acetylsalicylic acid and clopidogrel.

[4] the drug class of SSRIs consists of citalopram, escitalopram, duloxetine and the drug class of anticoagulants consists of apixaban.

[5] the drug class of SSRIs consists of citalopram, venlafaxine, duloxetine and the drug class of NSAIDs consists of ibuprofen.

[6] the drug class of SSRIs consists of citalopram, duloxetine, sertraline and the drug class of beta-blockers consist of metoprolol and its combination product

[7] the drug class of SSRIs consist of citalopram, duloxetine, venlafaxine, sertraline and the drug class of antidiabetics consists of metformin and sitagliptin.

[8] the drug class of serotonergic antidepressants consist of citalopram, duloxetine, venlafaxine, amitriptyline and the drug class of serotonergic opioids consists of oxycodone and tilidine and their combination products. Serotonin syndrome was not reported in reports explicitly related to these serotonergic opioids, thus, further statistical analysis could not be performed.
