# Supplementary material for: Analysis of drug-drug interactions in spontaneous adverse drug reaction reports from EudraVigilance focusing on psychiatric drugs and somatic medication
Source: BMC Psychiatry. 2025 Oct 2;25:914. doi: 10.1186/s12888-025-07352-8 (PMC12490046; doi:10.1186/s12888-025-07352-8)
Supplement: Supplementary file 6 — Supplementary Material 6. [file 12888_2025_7352_MOESM6_ESM.pdf]

Additional file 6) Analysis of the most frequently reported potentially interacting drug pairs grouped by drug classes and pharmacological effects in ADR reports from HCP and non-HCP.

Identification of ADR reports from HCP and non-HCP

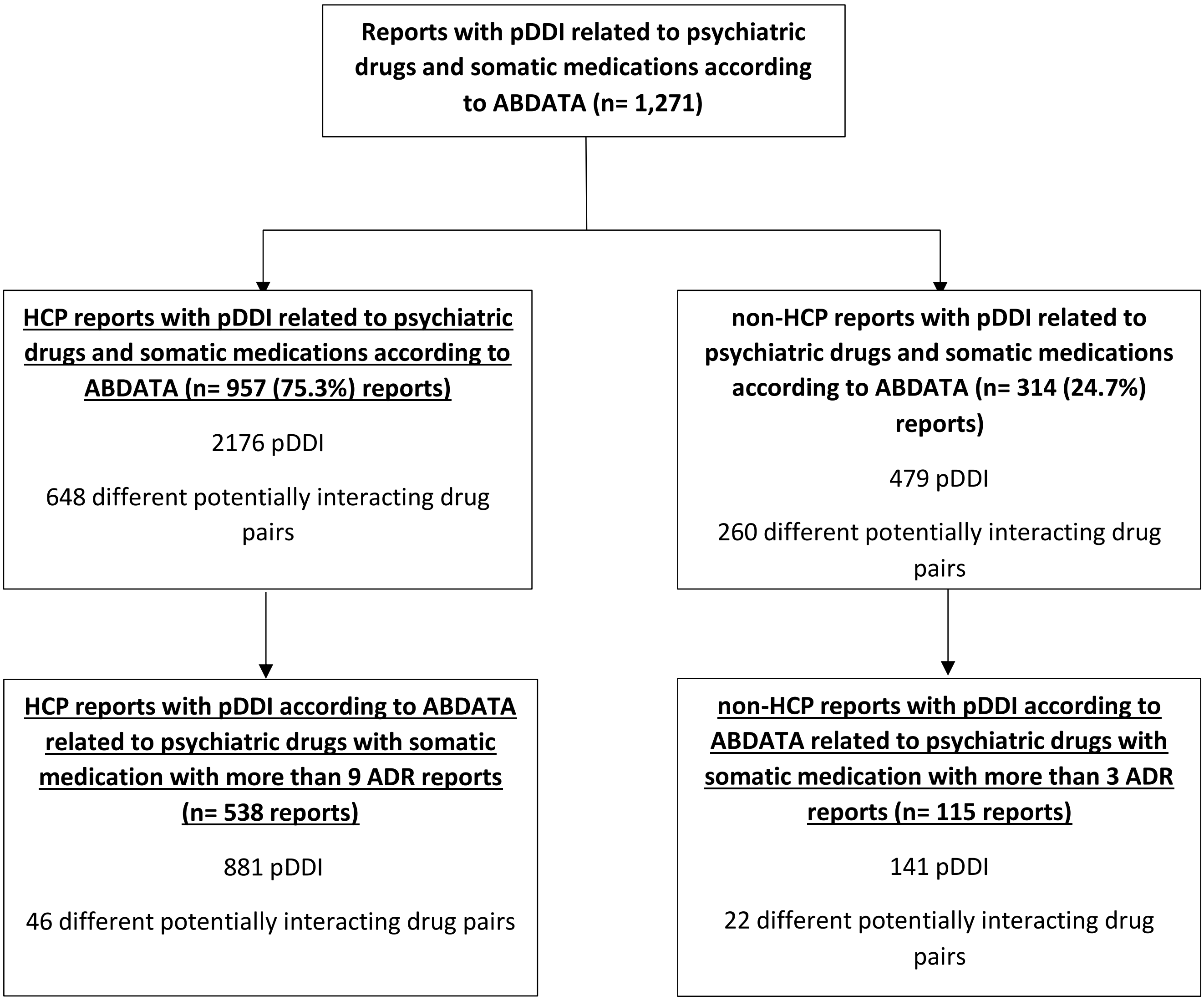

The most frequently reported pDDI with a proportion of more than 1% in the respective dataset were grouped by their pharmacological effects and drug classes. Thus, in ADR reports from HCP the

potentially interacting drug pairs had to be reported in at least 10 ADR reports and ADR reports from non-HCP in at least 4 ADR reports.

Additional file 6 Table 1) Most frequently reported pDDI grouped by pharmacological effects and drugs classes in ADR reports from HCP and non-HCP.

Rank	Reports from HCP of potentially interacting drug pairs with > 9 reports (n= 538 reports, 881 pDDI, 46 different potentially interacting drug pairs)	Rank	Reports from non-HCP of potentially interacting drug pairs with > 3 reports (n= 115 reports, 141 pDDI, 22 different potentially interacting drug pairs)
1.	Hyponatremia – antidepressants and antidiuretics (n= 252 (46.8%) reports, 322 (36.5%) pDDI)	1.	Bleeding events – SSRIs and platelet aggregation inhibitors or anticoagulants or NSAIDs (n= 64 (55.7%) reports, 66 (46.8%) pDDI)
2.	Bleeding events – SSRIs and platelet aggregation inhibitors or anticoagulants or NSAIDs (n= 188 (34.9%) reports, 208 (23.6%) pDDI)	2.	Hyponatremia – antidepressants and antidiuretics (n= 22 (19.1%) reports, 23 (16.3%) pDDI)
3.	Increased beta-blocker effects (hypotension, bradycardia) – SSRIs and beta-blockers (n= 106 (19.7%) reports, 108 (12.3%) pDDI)	3.	Hypo- or hyperglycemia – SSRIs and antidiabetics (n= 13 (11.3%) reports, 14 (9.9%) pDDI)
4.	Hypo- or hyperglycemia – SSRIs and antidiabetics (n= 56 (10.4%) reports, 57 (6.5%) pDDI)	4.	Increased beta-blocker effects (hypotension, bradycardia) – SSRIs and beta-blockers (n= 12 (10.4%) reports, 12 (8.5%) pDDI)

5.	Serotonin syndrome – serotonergic antidepressants and opiods (n= 39 (7.2%), 44 (5.0%) pDDI)	5.	Hyopthyreose – carbamazepine and levothyroxine (n= 7 (6.1%) reports, 7 (5.0%) pDDI)
6.	Granulocytopenia/agranulocyctosis – clozapine and ASS or ramipril or pantoprazole (n= 29 (5.4%), 33 (3.7%) pDDI)	6.	Serotonin syndrome – serotonergic antidepressants and opiods (n= 7 (6.1%) reports , 7 (5.0%) pDDI)
7.	Reduced efficacy of CYP substrates – metamizole and sertraline or valproinic acid (n= 30 (5.6%), 30 (3.4%) pDDI)	7.	Granulocytopenia/agranulocyctosis – clozapine and ASS or ramipril or pantoprazole (n= 4 (3.5%) reports, 4 (2.8%) pDDI)
8.	Increased anticholinergic effects – clozapine and pirenzepine (n= 25 (4.6%), 25 (2.8%) pDDI)	8.	Decreased efficacy of lamotrigine and hormonal contraceptive – lamotrigine and ethinylestradiol (n= 4 (3.5%) reports, 4 (2.8%) pDDI)
9.	Toxicity to valproinic acid and increased bleeding time – valproinic acid and acetylsalicylic acid (n= 22 (4.1%) reports 22 (2.5%) pDDI)	9.	Increased effects of PPI – fluoxetine and pantoprazole (n= 4 (3.5%) reports, 4 (2.8%) pDDI)
10.	Hyperammonic encephalopathy – valproinic aicd and topiramate (n= 12 (2.2%) reports, 12 (1.4%) pDDI)		
11.	Increased effects of citalopram – citalopram and omeprazole (n= 10 (1.9%), 10 (1.1%) pDDI)		

12.	Increased mortality in patients with dementia (n= 10 (1.9%), 10 (1.1%) pDDI)		

Additional file 6 Figure 1) The identified pDDI in the reports of the potentially interacting drug pairs from HCP according to ABDATA with more than 10 reports.

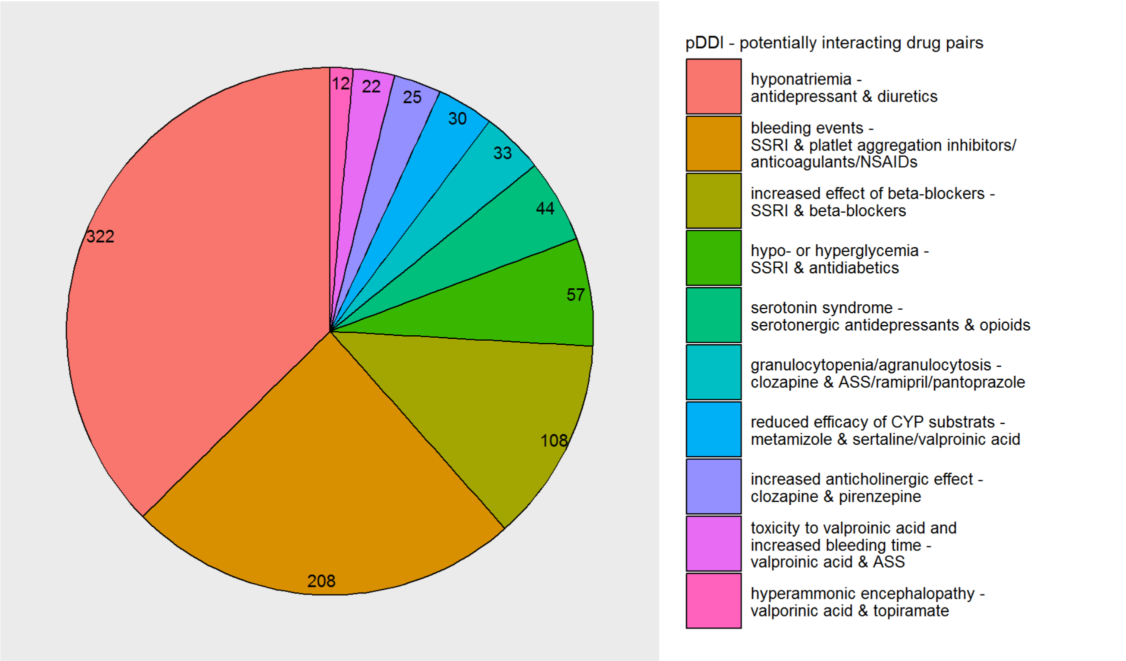

pDDI = potential drug-drug interactions; SSRI = selective serotonin reuptake inhibitors; NSAIDs = non-steroidal anti-inflammatory drugs; ASS = acetylsalicylic acid; CYP = cytochrome P450; HCP = health care professionals.

Additional file 6 Figure 1 shows the identified 791 pDDI (= pharmacological effects) according to ABDATA from 498 reports of 37 different potentially interacting drug pairs with more than 10 reports from HCP. The pDDI and the potentially interacting drug pairs were grouped by pharmacological effect and drug classes. Due to the grouping of drug classes, one report could be counted more than once per category. This is the case if more than one potentially interacting drug pair with the same ADR was reported (e.g. bleeding events related to anticoagulants and SSRIs and NSAIDs and SSRIs).

Only minor changes in the distribution and the number of reports were seen when the reports were counted once per category.
